# An Overview of the Genetic Variations of the SARS-CoV-2 Genomes Isolated in Southeast Asian Countries

**DOI:** 10.4014/jmb.2006.06009

**Published:** 2020-06-24

**Authors:** Polly Soo Xi Yap, Tse Siang Tan, Yoke Fun Chan, Kok Keng Tee, Adeeba Kamarulzaman, Cindy Shuan Ju Teh

**Affiliations:** 1 Department of Medical Microbiology, Faculty of Medicine, University of Malaya, 50603 Kuala Lumpur, Malaysia; 2 Department of Anaesthesiology and Intensive Care, UKM Medical Centre, 56000 Kuala Lumpur, Malaysia; 3 Department of Medicine, Faculty of Medicine, University of Malaya, 50603 Kuala Lumpur, Malaysia

**Keywords:** Coronavirus, SARS-CoV-2, genetic variation, mutation, COVID-19, Southeast Asia

## Abstract

Monitoring the mutation dynamics of human severe acute respiratory syndrome coronavirus 2 (SARS-CoV-2) is critical in understanding its infectivity, virulence and pathogenicity for development of a vaccine. In an “age of mobility,” the pandemic highlights the importance and vulnerability of regionalization and labor market interdependence in Southeast Asia. We intend to characterize the genetic variability of viral populations within the region to provide preliminary information for regional surveillance in the future. By analyzing 142 complete genomes from South East Asian (SEA) countries, we identified three central variants distinguished by nucleotide and amino acid changes.

## The Study

Human mobility (in parentheses) is one of the main factors that contribute to the worldwide dissemination of microorganisms. The spread of coronavirus disease 2019 (COVID-19) was recently reported to transmit to neighboring countries with relocation diffusion [1]. With most of the studies focusing on China, Western Europe and the USA, little is known about its evolution and genome variability in Southeast Asian (SEA) countries. SEA is home to more than half a billion or 9% of the world’s population. As the region grapples with a surge in infection cases since March 2020 [2], it is important to investigate purported mutations and the role of geographical proximity in shaping the genetic structure of the SARS-CoV-2 in SEA countries. On March 4, 2020, the World Health Organisation (WHO) outlined that only nine of the eleven countries have the capacity to test for COVID- 19 [3], suggesting that the lack of testing facilities could hinder the preparedness and response planning of these countries towards COVID-19.

Among the SEA countries, Malaysia, Thailand and Singapore employ a large number of migrant workers, with Malaysia being the top importer with approximately 2.23 million people [4]. Concurrently, there has been a mass exodus of Malaysians seeking greater economic security in Singapore, with approximately 450,000 people crossing the Malaysia-Singapore border daily [5]. The Indonesia authorities reported that more than 64,000 Indonesian migrant workers had returned from Malaysia amid the country’s ongoing lockdown [6]. Another type of human mobility is refugees. The political instability which holds sway in Myanmar has forced 10% of the population to emigrate in search of refuge [7]. More boats carrying Rohingya refugees were spotted off the coasts of Malaysia and southern Thailand, adding to the challenges faced by these countries fighting the pandemic outbreak [8]. This trend of massive internal mobility is expected to continue as the countries ease the lockdown in the foreseeable future, continuously shifting the genetic drift of the viral population. Studies have shown that human migration (gene flow) is a remarkable factor to consider in virus evolution [9]. Hence, characterization of the genetic variability of viral populations provides important insights in virus evolution and epidemiology for devising efficient and reliable infection control strategies. As of April 30, 2020, only 142 complete sequences (plural) of SARS-CoV-2 from six of the SEA countries, including Cambodia (*n* = 1), Malaysia (*n* = 16), the Philippines / Philippines (*n* = 12), Singapore (*n* = 74), Thailand (*n* = 31) and Vietnam (*n* = 8) (Supplementary Data 1) were deposited in the Global Initiative on Sharing Avian Influenza Data (GISAID) platform. All sequences have been included in the study. The majority of the strains were collected before their countries of origin imposed travel restrictions limiting entry (Supplementary Data 2). Therefore, in a data-limited situation, this study is intended to serve as an early snapshot of the genetic variations of the SARS-CoV-2 within the region and may help identify the endemic genotypes to provide fundamental information for regional surveillance in the future.

**Fig. 1:**
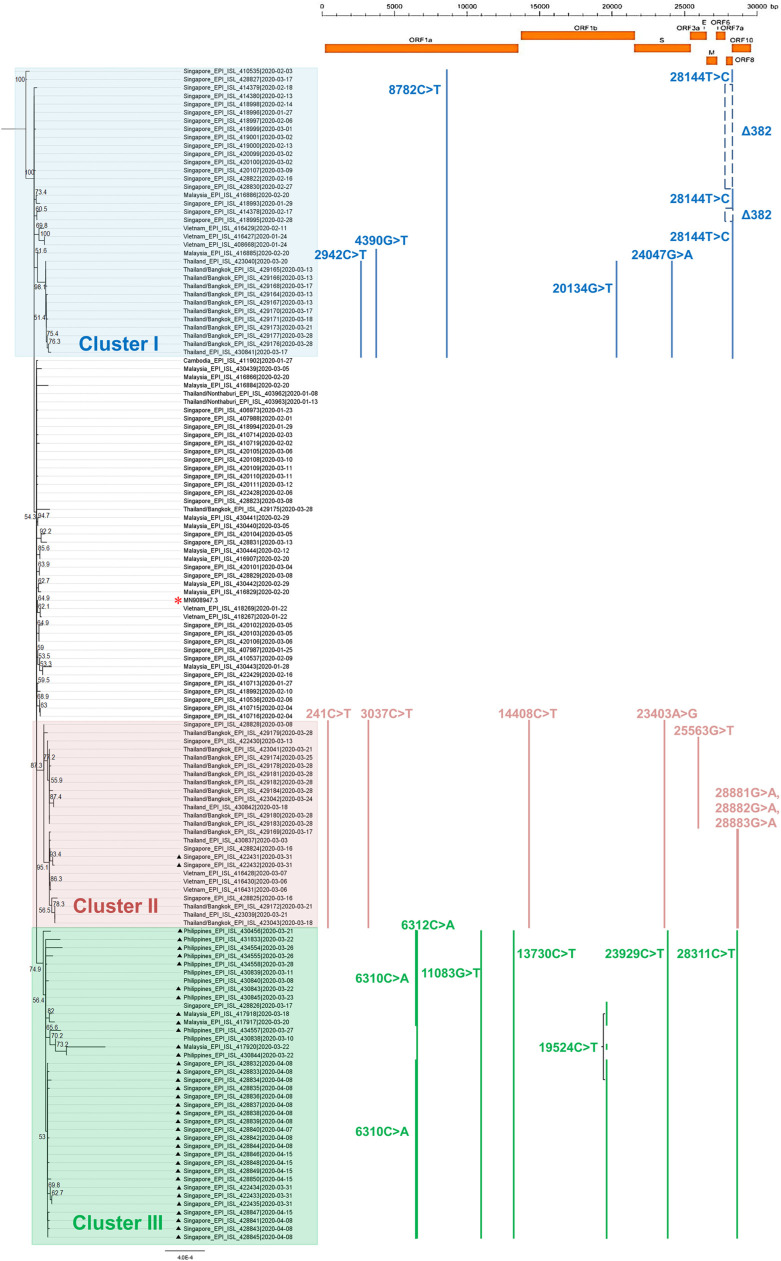
Phylogenetic tree inferred by using neighbor-joining method and Tamura-Nei model with 1,000 bootstrap replicates, representing complete SARS-CoV-2 genomes from SEA countries against the reference genome Wuhan-Hu-1 (*). Clusters identified were colored accordingly: Cluster I (blue), Cluster II (red) and Cluster III (green). Isolates with symbol (▲) denotes samples collected after country border control implementation whereas isolates without symbol denotes samples collected before country border control implementation.

**Fig. 2:**
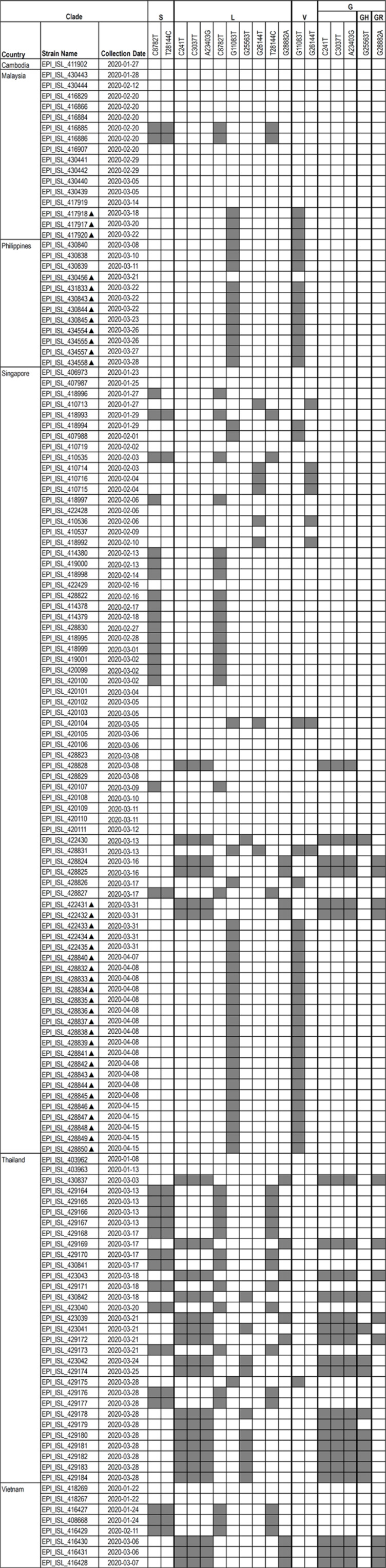
Heatmap showing the presence of marker variants of Clades S, L, V, G, GH and GR named by GISAID [12] in SEA SARS-CoV-2 genomes. Grey denotes the presence and empty space denotes the absence of the genes listed. Isolates with symbol ( ▲ ) denotes samples collected after country border control implementation whereas isolates without symbol denotes samples collected before country border control implementation.

Overall, all strains isolated before national implementation of border control were largely invariant (*n* = 43), while Clusters I, II, and III were sampled relatively recently and showed approximately 80% of synonymous mutations, suggesting possible ongoing adaptation of SARS-CoV-2 in the region. This is in agreement with a previous report in which local evolution of strains related to the mass gathering in Malaysia was observed [10]. We focused on mutations that have emerged multiple times and identified 22 recurrent mutations in the SEA SARS- CoV-2 genomes (Fig. 1). We also note that nearly 75% of the hits also overlap with candidate mutations, which may affect the phenotype of SARS-CoV-2 identified by van Dorp *et al.* [11]. There are 5 sites in the ORF1ab and 1 site each in the Spike and N protein that are characterized by a particularly large number of recurrent mutations (>20%). The GISAID SARS-CoV-2 portal [12] defined Clades S, G, and V, according to nucleotide variants that produce amino acid changes. These changes are located in ORF8 L84S (28144T>C); S protein D614G (23403A>G) and nsp3 G251V (26144G>T), respectively. We also mapped the strains with presence or absence of marker variants of larger clades named by GISAID [12] according to their geographical distributions (Fig. 2). Prior reports showed that the most common mutations were 8782C>T in ORF1ab and 28144T>C in ORF8 [13, 14]. ORF1ab is an orthologous gene with other human-associated betacoronaviruses, in particular SARS-CoV [15] and MERS-CoV [16], and was consistently identified as SARS-CoV-2 mutation hot spots [11]. Intriguingly, 92% of the strains isolated after country border control carried mutation at 11083G>T; otherwise this variant was hardly identified among strains before border control. Yeh & Contreras (Bull World Health Organ, https://www.who.int/bulletin/online_first/20-255752.pdf) reported that 11083G>T substitution was related to viral transmission among patients from a cruise with a 3-week quarantine period, and the study hypothesized that this mutation had taken place via RNA recombination with positive pressure. Further study is required to determine whether 11083G>T mutation plays a role in increasing the fitness in the carrier.

The current studied genomes showed phylogenetic relation with common recurrent mutations. Cluster I exhibited common recurrent mutation at 8782C>T in ORF1ab (*n* = 35). Forster *et al.* [17] observed that the ancestral S variant with these two mutations at 8782C>T and 28144T>C was predominantly identified in East Asia, but this variant outside of Asia was observed with striking, long mutational branch lengths. Instead of harboring the 28144T>C mutation, 15 Singaporean genomes from two different submitting laboratories were observed with a distinctive 382-nt deletion (Δ382) covering almost the entire ORF8. The variant was isolated between January 27 and March 9 of 2020, and was not identified in the subsequent isolates. As reported by Su *et al.* (bioRxiv, https://doi.org/10.1101/2020.03.11.987222), the Δ382 deletion removes the ORF8 transcription- regulatory sequence (TRS), resulted in enhanced downstream transcription of the N gene. Intriguingly, Malaysia closed its border on March 18, 2020, but the Δ382 has not been observed in its neighboring countries. As observed in other SARS-CoVs, mutations or deletions in ORF8 have been associated with viral replicative fitness in facilitating host adaption for interspecies transmission [18, 19]. Hence, core mutations (Δ382) in the S clade observed in the current study suggest an urgent need for comprehensive studies combining genomic, epidemiological and clinical data to understand the evolutionary pressure of this virus.

Cluster II was distinguished by not only the spike mutation D614G (23403A>G; G clade), but also at 241C>T, 3037C>T, and 14408C>T (*n* = 25). A further subcluster additionally containing mutations at 3 neighboring positions (28881G>A, 28882G>A, 28883G>A) in the N protein is also apparent within this clade. The G variant was rarely sampled in Asia but corresponded / corresponded to the most frequent variant in Europe [12]. However, there is no evidence that the increasing predominance of this mutation was caused by convergent selection or bottleneck events. In the current study, G variants were identified in strains predominantly from Thailand, followed by Singapore and Vietnam. Cluster III belonged to clades outside the reported S, G and V clades, and was distinguished by shared mutations at 6312C>A, 11083G>T, 13730C>T and 19524C>T in ORF1ab, 23929C>T in spike and 28311C>T in the N protein (*n* = 38). This variant was beginning to be observed in strains from Malaysia, the Philippines and Singapore from mid-March onwards. On April 19, 2020, a new cluster emerged from students returning to Malaysia from Indonesia [20]. Therefore, it will be valuable to include strains from Indonesia as well as other SEA countries in the future for endemic genetic variation surveillance. Similar to other SARS-CoVs, the spike protein and its host receptor have been the key targets for drug and vaccine development because the protein is relatively more conserved and is critical for viral infection [21]. In the current study, each of the clusters was identified with one mutation at the spike protein. Further analysis with PROVEAN [22] prediction of the amino acid variants showed that mutations at 23403A>G as well as 23929C>T in the spike protein were deleterious/damaging to protein function (PROVEAN score ≤ -2.5) (Supplementary Figure). Notably, both spike mutations were observed only from March 8th onwards, suggesting the emergence of positive selection. All Philippine strains carried 23929C>T mutations although this mutation was observed only after border control was implemented in Malaysia and Singapore. Nevertheless, the impact of these mutations towards the virus affinity to host receptor remains to be elucidated. There has been a lack of association between genotypes and clinical presentations of COVID-19 [23]. Hence, this characterization of SARS-CoV-2 variants could lead to better treatment strategies in the future.

The evolution and transmission of the SARS-CoV-2 is potentially affected by distinctive travel histories, founder events, host characteristics as well as geographical and climate factors. It is prudent to consider the possibility that mutational variants might influence the virus spread and subsequently the clinical presentation and outcome. Therefore, the described core mutations and phylogenetic classification in this work may provide information regarding outbreak control as well as evaluating the clinical and epidemiological outcomes of SARS- CoV-2 infection.

## Methodology

A phylogenetic tree was constructed using 142 complete genome sequences from the South East Asia countries plus the reference genome Wuhan-Hu-1 (MN908947.3). The SARS-CoV-2 genomes were obtained from the GISAID (https://www.gisaid.org/), as of April 30, 2020. Genome sequence alignment was performed using Multiple Sequence Comparison by Log-Expectation (MUSCLE) and the phylogenetic tree was inferred by using the neighbor-joining method and Tamura-Nei model with 1,000 bootstrap replicates in Geneious software (version 2020.1; Biomatters Ltd.). The sequences were also aligned to the reference genome. Sites were masked in the first 130bp and last 50 bp, as were other ambiguous positions following the protocol advocated by van Dorp *et al.* [11] and De Maio *et al.* (http://virological.org/t/issues-with-sars-cov-2-sequencing-data/473, accessed May 12, 2020). Estimation of the best fitting substitutions in nucleotide and amino acid were also detected within Geneious. We only considered variants at a site if present with frequency ≥5% [24, 25].

## Data Availability

The genome sequences of the SARS-CoV-2 used in this study are available from the GISAID database (https://www.gisaid.org/), upon registration.

## Supplemental Materials



Supplementary data for this paper are available on-line only at http://jmb.or.kr.
