# Supplementary material for: An Overview of the Genetic Variations of the SARS-CoV-2 Genomes Isolated in Southeast Asian Countries
Source: J Microbiol Biotechnol. 2020 Jun 24;30(7):962–6. doi: 10.4014/jmb.2006.06009 (PMC9728395; doi:10.4014/jmb.2006.06009)
Supplement: Supplementary file 1 [file JMB-30-7-962-supple.pdf]

J. Microbiol. Biotechnol.
https://doi.org/10.4014/jmb.2006.06009

Supplementary Data 1. Acknowledgements to Sequencing laboratories and GISAID Datat

We gratefully acknowledge the authors, originating and submitting laboratories of the sequences from GISAID's EpiFlu™ Database on which this research is based. The list is detailec
All submitters of data may be contacted directly vwww.gisaid.or

Accession ID	Virus name	Location	Collection date	Originating lab	Submitting lab	Authors
MN908947 (NCBI)	Severe acute respiratory syndrome coronav	Asia / China / Hubei / Wuhan	December' 2019	Shanghai Public Health Clinical Center & School of Public Health, Fud	Shanghai Public Health Clinical Center & School of Public He	Wu,F., Zhao,S., Yu,B., Chen,Y.-M., Wang,W., Hu,Y., Song,Z.-G., Tao,Z.-W., Tian,J.-H., Pei,Y.-Y., Yuan,M.L., Zhang,Y.-L., Dai,F.-H., Liu,Y., Wang,Q.-M., Zheng,J.-J., Xu,L., Holmes,E.C. and Zhang,Y.-Z.
EPI_ISL_411902	hCoV-19/Cambodia/0012/2020	Asia / Cambodia / Sihanoukville	2020-01-27	Virology Unit, Institut Pasteur du Cambodge.	Virology Unit, Institut Pasteur du Cambodge (Sequencing of by: Jessica E Manning/Jennifer A Bohl at Malaria and Vector Research Research Laboratory, National Institute of Allergy and Infectious Diseases and Vida Ahyong from Chan-Zuckerberg	Erik A Karlsson, Jennifer A Bohl, Vida Ahyong, Veasna Duong, Philippe Dussart, Jessica E Manning.
EPI_ISL_410487	hCoV-19/Malaysia/CL5045/2020	Asia / Malaysia	2020-01-24	unknown	National Public Health Laboratory	Yu Kie,C., Norazimah,T., Rehan Shuhada,A.B., Selvanesan,S., Noorliza,M.N. and Hani,M.H.
EPI_ISL_410488	hCoV-19/Malaysia/CL5047/2020	Asia / Malaysia	2020-01-24	unknown	National Public Health Laboratory	Yu Kie,C., Norazimah,T., Rehan Shuhada,A.B., Selvanesan,S., Noorliza,M.N. and Hani,M.H.
EPI_ISL_410489	hCoV-19/Malaysia/CL5049/2020	Asia / Malaysia	2020-01-24	unknown	National Public Health Laboratory	Yu Kie,C., Norazimah,T., Rehan Shuhada,A.B., Selvanesan,S., Noorliza,M.N. and Hani,M.H.
EPI_ISL_416829	hCoV-19/Malaysia/MKAK-CL-2020-5045/2020	Asia / Malaysia / Selangor	2020-02-20	National Public Health Laboratory	Malaysia Genome Institute	Mohd Noor Mat Isa, Irni Suhayu Sapian, Yusuf Muhammd Noor, Hezreen Iqbal, Mohd Faizal Abu Bakar, Enizza Kassim, Shamsidar Sopie, Azrin Ahmad, Norazfa Johari, Norazimah Tajudin, Selvanesan Sengol, Yu Kie Chem, Hani Mat Hussin, Shahrul Hisham Zainal Ariffin
EPI_ISL_416866	hCoV-19/Malaysia/MKAK-CL-2020-5047/2020	Asia/ Malaysia/Selangor	2020-02-20	National Public Health Laboratory	Malaysia Genome Institute	Mohd Noor Mat Isa, Irni Suhayu Sapian, Yusuf Muhammd Noor, Hezreen Iqbal, Mohd Faizal Abu Bakar, Enizza Kassim, Shamsidar Sopie, Azrin Ahmad, Norazfa Johari, Norazimah Tajudin, Selvanesan Sengol, Yu Kie Chem, Hani Mat Hussin, Shahrul Hisham Zainal Ariffin
EPI_ISL_416884	hCoV-19/Malaysia/MKAK-CL-2020-5049/2020	Asia / Malaysia / Selangor	2020-02-20	National Public Health Laboratory	Malaysia Genome Institute	Mohd Noor Mat Isa, Irni Suhayu Sapian, Yusuf Muhammd Noor, Hezreen Iqbal, Mohd Faizal Abu Bakar, Enizza Kassim, Shamsidar Sopie, Azrin Ahmad, Norazfa Johari, Norazimah Tajudin, Selvanesan Sengol, Yu Kie Chem, Hani Mat Hussin, Shahrul Hisham Zainal Ariffin
EPI_ISL_416885	hCoV-19/Malaysia/MKAK-CL-2020-5096/2020	Asia/ Malaysia/Selangor	2020-02-20	National Public Health Laboratory	Malaysia Genome Institute	Mohd Noor Mat Isa, Irni Suhayu Sapian, Yusuf Muhammd Noor, Hezreen Iqbal, Mohd Faizal Abu Bakar, Enizza Kassim, Shamsidar Sopie, Azrin Ahmad, Norazfa Johari, Norazimah Tajudin, Selvanesan Sengol, Yu Kie Chem, Hani Mat Hussin, Shahrul Hisham Zainal Ariffin
EPI_ISL_416886	hCoV-19/Malaysia/MKAK-CL-2020-6430/2020	Asia / Malaysia / Selangor	2020-02-20	National Public Health Laboratory	Malaysia Genome Institute	Mohd Noor Mat Isa, Irni Suhayu Sapian, Yusuf Muhammd Noor, Hezreen Iqbal, Mohd Faizal Abu Bakar, Enizza Kassim, Shamsidar Sopie, Azrin Ahmad, Norazfa Johari, Norazimah Tajudin, Selvanesan Sengol, Yu Kie Chem, Hani Mat Hussin, Shahrul Hisham Zainal Ariffin
EPI_ISL_416907	hCoV-19/Malaysia/MKAK-CL-2020-7554/2020	Asia/ Malaysia/Selangor	2020-02-20	National Public Health Laboratory	Malaysia Genome Institute	Mohd Noor Mat Isa, Irni Suhayu Sapian, Yusuf Muhammd Noor, Hezreen Iqbal, Mohd Faizal Abu Bakar, Enizza Kassim, Shamsidar Sopie, Azrin Ahmad, Norazfa Johari, Norazimah Tajudin, Selvanesan Sengol, Yu Kie Chem, Hani Mat Hussin, Shahrul Hisham Zainal Ariffin
EPI_ISL_417917	hCoV-19/Malaysia/189332/2020	Asia / Malaysia / Kuala Lumpur	2020-03-20	Department of Medical Microbiology, University Malaya Medical Centre	Department of Medical Microbiology	Yoong Min CHONG, Sasheela PONNAMPALAVANAR, Sharifah Faridah SYED OMAR, Adeeba KAMARULZAMAN,Vijayan MUNUSAMY, Chee Kuan WONG, Cindy Shuan Ju TEH, I-Ching SAM, Yoke Fun Chan, University Malaya Medical Centre COVID Team
EPI_ISL_417918	hCoV-19/Malaysia/188407/2020	Asia / Malaysia / Kuala Lumpur	2020-03-18	Department of Medical Microbiology, University Malaya Medical Centre	Department of Medical Microbiology, Faculty of Medicine, Uni	Yoong Min CHONG, Sasheela PONNAMPALAVANAR, Sharifah Faridah SYED OMAR, Adeeba KAMARULZAMAN,Vijayan MUNUSAMY, Chee Kuan WONG, Cindy Shuan Ju TEH, I-Ching SAM, Yoke Fun Chan, University Malaya Medical Centre COVID Team
EPI_ISL_417919	hCoV-19/Malaysia/186197/2020	Asia / Malaysia / Kuala Lumpur	2020-03-14	Department of Medical Microbiology, University Malaya Medical Centre	Department of Medical Microbiology, Faculty of Medicine, Uni	Yoong Min CHONG, Sasheela PONNAMPALAVANAR, Sharifah Faridah SYED OMAR, Adeeba KAMARULZAMAN,Vijayan MUNUSAMY, Chee Kuan WONG, Fadhil Hadi JAMALUDDIN, Han Ming GAN, Cindy Shuan Ju TEH, I-Ching SAM, Yoke Fun CHAN, University Malaya Medical Centre COVID Team
EPI_ISL_417920	hCoV-19/Malaysia/190300/2020	Asia / Malaysia / Kuala Lumpur	2020-03-22	Department of Medical Microbiology, University Malaya Medical Centre	Department of Medical Microbiology, Faculty of Medicine, Uni	Yoong Min CHONG, Sasheela PONNAMPALAVANAR, Sharifah Faridah SYED OMAR, Adeeba KAMARULZAMAN,Vijayan MUNUSAMY, Chee Kuan WONG, Fadhil Hadi JAMALUDDIN, Cindy Shuan Ju TEH, I-Ching SAM, Yoke Fun Chan, University Malaya Medical Centre COVID Team
EPI_ISL_430439	hCoV-19/Malaysia/IMR_WC1177/2020	Asia / Malaysia	2020-03-05	Institute for Medical Research, Infectious Disease Research Centre, I	Institute for Medical Research Infectious Disease Research C	Suppiah.J, Mohd-Zawawi.Z, Kalyanasundram.J, Azizan.M-A, Mat-Sharani.S, Hisham.H-A, Tan.L-P, Abdul-Wahid.M-Z, Mohd-Zain.R, Ahmad.N, Thayan.R
EPI_ISL_430440	hCoV-19/Malaysia/IMR_WC1170/2020	Asia / Malaysia	2020-03-05	Institute for Medical Research, Infectious Disease Research Centre, N	Institute for Medical Research, Infectious Disease Research C	Suppiah.J, Mohd-Zawawi.Z, Kalyanasundram.J, Azizan.M-A, Mat-Sharani.S, Hisham.H-A, Tan.L-P, Abdul-Wahid.M-Z, Tengku-Abd-Rashid.T-R, Mohd-Zain.R, Ahmad.N, Thayan.R
EPI_ISL_430441	hCoV-19/Malaysia/IMR_WC1097/2020	Malaysia	2020-02-29	Institute for Medical Research, Infectious Disease Research Centre, N	Institute for Medical Research, Infectious Disease Research C	Suppiah.J, Mohd-Zawawi.Z, Kalyanasundram.J, Azizan.M-A, Mat-Sharani.S, Hisham.H-A, Tan.L-P, Abdul-Wahid.M-Z, Tengku-Rogayah.TAR, Mohd-Zain.R, Ahmad.N, Thayan.R
EPI_ISL_430442	hCoV-19/Malaysia/IMR_WC1098/2020	Asia / Malaysia	2020-02-29	Institute for Medical Research, Infectious Disease Research Centre, N	Institute for Medical Research, Infectious Disease Research C	Suppiah.J, Mohd-Zawawi.Z, Kalyanasundram.J, Azizan.M-A, Mat-Sharani.S, Hisham.H-A, Tan.L-P, Abdul-Wahid.M-Z, Tengku-Rogayah.TAR, Mohd-Zain.R, Ahmad.N, Thayan.R
EPI_ISL_430443	hCoV-19/Malaysia/IMR_WC085/2020	Asia / Malaysia	2020-01-28	Institute for Medical Research, Infectious Disease Research Centre, N	Institute for Medical Research, Infectious Disease Research C	Suppiah.J, Mohd-Zawawi.Z, Kalyanasundram.J, Azizan.M-A, Mat-Sharani.S, Hisham.H-A, Tan.L-P, Abdul-Wahid.M-Z, Tengku-Rogayah.TAR, Mohd-Zain.R, Ahmad.N, Thayan.R
EPI_ISL_430444	hCoV-19/Malaysia/IMR_WC627/2020	Asia / Malaysia	2020-02-12	Institute for Medical Research, Infectious Disease Research Centre, N	Institute for Medical Research, Infectious Disease Research C	Suppiah.J, Mohd-Zawawi.Z, Kalyanasundram.J, Azizan.M-A, Mat-Sharani.S, Hisham.H-A, Tan.L-P, Abdul-Wahid.M-Z, Tengku-Rogayah.TAR, Mohd-Zain.R, Ahmad.N, Thayan.R
EPI_ISL_410302	hCoV-19/Philippines/026/2020	Asia / Philippines	2020-01-26	unknown	Amalea Dulcene Nicolasora Research Institute for Tropical M	Nicolasora,A.D., Mercado,E.S., Polotan,F.M., Manalo,J.G., Medado,I.P., Tujan,M.A., Onza,O.T. and Cruz,K.M.
EPI_ISL_410314	hCoV-19/Philippines/026N/2020	Asia / Philippines	2020-02-06	unknown	Joanna Ina Manalo Research Institute for Tropical Medicine, I	Manalo,J.I. and Nicolasora,A.D.
EPI_ISL_410344	hCoV-19/Philippines/025N/2020	Asia / Philippines	2020-01-26	unknown	Joanna Ina Manalo Research Institute for Tropical Medicine, I	Mercado,E.S., Manalo,J.I., Nicolasora,A.D., Medado,I.P., Tujan,M.A., Onza,O.T., Cruz,K.M. and Polotan,F.M.
EPI_ISL_410345	hCoV-19/Philippines/022N/2020	Asia / Philippines	2020-01-23	unknown	Joanna Ina Manalo Research Institute for Tropical Medicine, I	Mercado,E.S., Manalo,J.I., Nicolasora,A.D., Medado,I.P., Tujan,M.A., Onza,O.T., Cruz,K.M. and Polotan,F.M.
EPI_ISL_430456	hCoV-19/Philippines/RITM-03/2020	Asia / Philippines	2020-03-21	Rizal Medical Center	Research Institute for Tropical Medicine	Medado,I.A.P., Bautista,C.T., Onza,O.J.T., Polotan,F.G.M., Mercado,E.S.
EPI_ISL_430838	hCoV-19/Philippines/RITM-01/2020	Asia / Philippines	2020-03-10	Makati Medical Center	Research Institute for Tropical Medicine	Medado,I.A.P., Bautista,C.T., Onza,O.J.T., Polotan,F.G.M., Brunker, K., Mercado,E.S., Manalo, D.L., Demetria, C.S.
EPI_ISL_430839	hCoV-19/Philippines/RITM-02/2020	Asia / Philippines	2020-03-11	Research Institute for Tropical Medicine	Research Institute for Tropical Medicine	Medado,I.A.P., Bautista,C.T., Onza,O.J.T., Polotan,F.G.M., Brunker, K., Mercado,E.S., Manalo, D.L., Demetria, C.S.
EPI_ISL_430840	hCoV-19/Philippines/RITM-04/2020	Asia / Philippines	2020-03-08	Veterans Memorial Medical Center	Research Institute for Tropical Medicine	Medado,I.A.P., Bautista,C.T., Onza,O.J.T., Polotan,F.G.M., Brunker, K., Mercado,E.S., Manalo, D.L., Demetria, C.S.
EPI_ISL_430843	hCoV-19/Philippines/RITM-05/2020	Asia / Philippines	2020-03-22	Bethany Hospital	Research Institute for Tropical Medicine	Medado,I.A.P., Bautista,C.T., Onza,O.J.T., Polotan,F.G.M., Brunker, K., Mercado,E.S., Manalo, D.L., Demetria, C.S.
EPI_ISL_430844	hCoV-19/Philippines/RITM-06/2020	Asia / Philippines	2020-03-22	Lung Center of the Philippines	Research Institute for Tropical Medicine	Medado,I.A.P., Bautista,C.T., Onza,O.J.T., Polotan,F.G.M., Brunker, K., Mercado,E.S., Manalo, D.L., Demetria, C.S.
EPI_ISL_430845	hCoV-19/Philippines/RITM-07/2020	Asia / Philippines	2020-03-23	Pasig City General Hospital	Research Institute for Tropical Medicine	Medado,I.A.P., Bautista,C.T., Onza,O.J.T., Polotan,F.G.M., Brunker, K., Mercado,E.S., Manalo, D.L., Demetria, C.S.
EPI_ISL_431833	hCoV-19/Philippines/PGC001/2020	Asia / Philippines / Manila	2020-03-22	National Institutes of Health, University of the Philippines Manila	Philippine Genome Center, University of the Philippines System	Carlo M. Lapid, Francis A. Tablizo, Benedict A. Maralit, Jan Michael C. Yap, Raul V. Destura, Marissa M. Alejandria, El King D. Morado, Joshua Gregor A. Dizon, Jo-Hannah S. Llames, Shiela Mae M. Araiza, Kris P. Punayan, Kristianne Arielle D. Gabriel, Shebna Rose D. Fabilloren, Shana F. Genavia, Jarvin E. Nipales, Alessandra C. Sanchez, Haifa L.Gaza, Joy Ann Petronio-Santos, Julius Aaron Mejia, Maribell Dollete, Sonia Salamat, Christina Tan, Bernard Demot, John Mark Velasco, Ev
EPI_ISL_434554	hCoV-19/Philippines/PGC002/2020	Asia / Philippines / Manila	2020-03-26	National Institutes of Health, University of the Philippines Manila	Philippine Genome Center	Carlo M. Lapid, Francis A. Tablizo, Benedict A. Maralit, Jan Michael C. Yap, Raul V. Destura, Marissa M. Alejandria, El King D. Morado, Joshua Gregor A. Dizon, Jo-Hannah S. Llames, Shiela Mae M. Araiza, Kris P. Punayan, Kristianne Arielle D. Gabriel, Shebna Rose D. Fabilloren, Shana F. Genavia, Jarvin E. Nipales, Alessandra C. Sanchez, Haifa L.Gaza, Joy Ann Petronio-Santos, Julius Aaron Mejia, Maribell Dollete, Sonia Salamat, Christina Tan, Bernard Demot, John Mark Velasco, Ev
EPI_ISL_434555	hCoV-19/Philippines/PGC003/2020	Asia / Philippines / Manila	2020-03-26	National Institutes of Health, University of the Philippines Manila	Philippine Genome Center	Carlo M. Lapid, Francis A. Tablizo, Benedict A. Maralit, Jan Michael C. Yap, Raul V. Destura, Marissa M. Alejandria, El King D. Morado, Joshua Gregor A. Dizon, Jo-Hannah S. Llames, Shiela Mae M. Araiza, Kris P. Punayan, Kristianne Arielle D. Gabriel, Shebna Rose D. Fabilloren, Shana F. Genavia, Jarvin E. Nipales, Alessandra C. Sanchez, Haifa L.Gaza, Joy Ann Petronio-Santos, Julius Aaron Mejia, Maribell Dollete, Sonia Salamat, Christina Tan, Bernard Demot, John Mark Velasco, Ev
EPI_ISL_434557	hCoV-19/Philippines/PGC005/2020	Asia / Philippines / Manila	2020-03-27	National Institutes of Health, University of the Philippines Manila	Philippine Genome Center	Carlo M. Lapid, Francis A. Tablizo, Benedict A. Maralit, Jan Michael C. Yap, Raul V. Destura, Marissa M. Alejandria, El King D. Morado, Joshua Gregor A. Dizon, Jo-Hannah S. Llames, Shiela Mae M. Araiza, Kris P. Punayan, Kristianne Arielle D. Gabriel, Shebna Rose D. Fabilloren, Shana F. Genavia, Jarvin E. Nipales, Alessandra C. Sanchez, Haifa L.Gaza, Joy Ann Petronio-Santos, Julius Aaron Mejia, Maribell Dollete, Sonia Salamat, Christina Tan, Bernard Demot, John Mark Velasco, Ev
EPI_ISL_434558	hCoV-19/Philippines/PGC006/2020	Asia / Philippines / Manila	2020-03-28	National Institutes of Health, University of the Philippines Manila	Philippine Genome Center	Carlo M. Lapid, Francis A. Tablizo, Benedict A. Maralit, Jan Michael C. Yap, Raul V. Destura, Marissa M. Alejandria, El King D. Morado, Joshua Gregor A. Dizon, Jo-Hannah S. Llames, Shiela Mae M. Araiza, Kris P. Punayan, Kristianne Arielle D. Gabriel, Shebna Rose D. Fabilloren, Shana F. Genavia, Jarvin E. Nipales, Alessandra C. Sanchez, Haifa L.Gaza, Joy Ann Petronio-Santos, Julius Aaron Mejia, Maribell Dollete, Sonia Salamat, Christina Tan, Bernard Demot, John Mark Velasco, Ev
EPI_ISL_406973	hCoV-19/Singapore/1/2020	Asia / Singapore	2020-01-23	Singapore General Hospital	National Public Health Laboratory	Mak, TM; Octavia S; Chavatte JM; Zhou, ZY; Cui, L; Lin, RTP
EPI_ISL_407987	hCoV-19/Singapore/2/2020	Asia / Singapore	2020-01-25	Singapore General Hospital	Programme in Emerging Infectious Diseases, Duke-NUS Med	Danielle E Anderson, Martin Linster, Yan Zhuang, Jayanthi Jayakumar, Kian Sing Chan, Lynette LE Oon, Jenny GH Low, Yvonne CF Su, Linfa Wang, Gavin JD Smith
EPI_ISL_407988	hCoV-19/Singapore/3/2020	Asia / Singapore	2020-02-01	National Centre for Infectious Diseases	Programme in Emerging Infectious Diseases, Duke-NUS Med	Danielle E Anderson, Martin Linster, Yan Zhuang, Jayanthi Jayakumar, David CB Lye, Yee Sin Leo, Barnaby E Young, Yvonne CF Su, Linfa Wang, Gavin JD Smith
EPI_ISL_410535	hCoV-19/Singapore/4/2020	Asia / Singapore	2020-02-03	National Centre for Infectious Diseases	Programme in Emerging Infectious Diseases, Duke-NUS Med	Danielle E Anderson, Martin Linster, Yan Zhuang, Jayanthi Jayakumar, David CB Lye, Yee Sin Leo, Barnaby E Young, Yvonne CF Su, Gavin JD Smith
EPI_ISL_410536	hCoV-19/Singapore/5/2020	Asia / Singapore	2020-02-06	Singapore General Hospital, Molecular Laboratory, Division of Pathology	Programme in Emerging Infectious Diseases, Duke-NUS Med	Danielle E Anderson, Martin Linster, Yan Zhuang, Jayanthi Jayakumar, Kian Sing Chan, Lynette LE Oon, Shirin Kalimuddin, Jenny GH Low, Yvonne CF Su, Gavin JD Smith
EPI_ISL_410537	hCoV-19/Singapore/6/2020	Asia / Singapore	2020-02-09	Singapore General Hospital, Molecular Laboratory, Division of Pathology	Programme in Emerging Infectious Diseases, Duke-NUS Med	Danielle E Anderson, Martin Linster, Yan Zhuang, Jayanthi Jayakumar, Kian Sing Chan, Lynette LE Oon, Shirin Kalimuddin, Jenny GH Low, Yvonne CF Su, Gavin JD Smith
EPI_ISL_410713	hCoV-19/Singapore/7/2020	Asia / Singapore	2020-01-27	National Public Health Laboratory, National Centre for Infectious Diseas	National Public Health Laboratory, National Centre for Infectio	Octavia S, Mak TM, Cui L, Lin RTP
EPI_ISL_410714	hCoV-19/Singapore/8/2020	Asia / Singapore	2020-02-03	National Public Health Laboratory, National Centre for Infectious Diseas	National Public Health Laboratory, National Centre for Infectio	Octavia S, Mak TM, Cui L, Lin RTP
EPI_ISL_410715	hCoV-19/Singapore/9/2020	Asia / Singapore	2020-02-04	National Public Health Laboratory, National Centre for Infectious Disea	National Public Health Laboratory, National Centre for Infectio	Octavia S, Mak TM, Cui L, Lin RTP
EPI_ISL_410716	hCoV-19/Singapore/10/2020	Asia / Singapore	2020-02-04	National Public Health Laboratory, National Centre for Infectious Disea	National Centre for Infectious Diseases, National Centre for Ir	Octavia S, Mak TM, Cui L, Lin RTP
EPI_ISL_410719	hCoV-19/Singapore/11/2020	Asia / Singapore	2020-02-02	National Public Health Laboratory	National Public Health Laboratory	Octavia S, Mak TM, Cui L, Lin RTP
EPI_ISL_414378	hCoV-19/Singapore/12/2020	Asia / Singapore	2020-02-17	National Centre for Infectious Diseases	Programme in Emerging Infectious Diseases, Duke-NUS Med	Danielle E Anderson, Martin Linster, Yan Zhuang, Jayanthi Jayakumar, Louisa Sun, David CB Lye, Yee Sin Leo, Barnaby E Young, Yvonne CF Su, Gavin JD Smith
EPI_ISL_414379	hCoV-19/Singapore/13/2020	Asia / Singapore	2020-02-18	National Centre for Infectious Diseases	Programme in Emerging Infectious Diseases, Duke-NUS Med	Danielle E Anderson, Martin Linster, Yan Zhuang, Jayanthi Jayakumar, David CB Lye, Yee Sin Leo, Barnaby E Young, Yvonne CF Su, Gavin JD Smith
EPI_ISL_414380	hCoV-19/Singapore/14/2020	Asia / Singapore	2020-02-13	National Centre for Infectious Diseases	Programme in Emerging Infectious Diseases, Duke-NUS Med	Danielle E Anderson, Martin Linster, Yan Zhuang, Jayanthi Jayakumar, David CB Lye, Yee Sin Leo, Barnaby E Young, Yvonne CF Su, Gavin JD Smith
EPI_ISL_418992	hCoV-19/Singapore/17/2020	Asia / Singapore	2020-02-10	National Public Health Laboratory, National Centre for Infectious Diseas	National Public Health Laboratory, National Centre for Infectio	Mak TM, Octavia S, Cui L, Lin RTP
EPI_ISL_418993	hCoV-19/Singapore/20/2020	Asia / Singapore	2020-01-29	National Public Health Laboratory, National Centre for Infectious Diseas	National Public Health Laboratory, National Centre for Infectio	Mak TM, Octavia S, Cui L, Lin RTP
EPI_ISL_418994	hCoV-19/Singapore/3Clin/2020	Asia / Singapore	2020-01-29	National Public Health Laboratory, National Centre for Infectious Diseas	National Public Health Laboratory, National Centre for Infectio	Mak TM, Octavia S, Cui L, Lin RTP
EPI_ISL_418995	hCoV-19/Singapore/12Clin/2020	Asia / Singapore	2020-02-28	National Public Health Laboratory, National Centre for Infectious Diseas	National Public Health Laboratory, National Centre for Infectio	Mak TM, Octavia S, Cui L, Lin RTP
EPI_ISL_418996	hCoV-19/Singapore/15/2020	Asia / Singapore	2020-01-27	National Public Health Laboratory, National Centre for Infectious Diseas	National Public Health Laboratory, National Centre for Infectio	Mak TM, Octavia S, Cui L, Lin RTP
EPI_ISL_418997	hCoV-19/Singapore/16/2020	Asia / Singapore	2020-02-06	National Public Health Laboratory, National Centre for Infectious Diseas	National Public Health Laboratory, National Centre for Infectio	Mak TM, Octavia S, Cui L, Lin RTP
EPI_ISL_418998	hCoV-19/Singapore/14Clin/2020	Asia / Singapore	2020-02-14	National Public Health Laboratory, National Centre for Infectious Diseas	National Public Health Laboratory, National Centre for Infectio	Mak TM, Octavia S, Cui L, Lin RTP
EPI_ISL_418999	hCoV-19/Singapore/18/2020	Asia / Singapore	2020-03-01	National Public Health Laboratory, National Centre for Infectious Diseas	National Public Health Laboratory, National Centre for Infectio	Mak TM, Octavia S, Cui L, Lin RTP
EPI_ISL_419000	hCoV-19/Singapore/21/2020	Asia / Singapore	2020-02-13	National Public Health Laboratory, National Centre for Infectious Diseas	National Public Health Laboratory, National Centre for Infectio	Mak TM, Octavia S, Cui L, Lin RTP
EPI_ISL_419001	hCoV-19/Singapore/19/2020	Asia / Singapore	2020-03-02	National Public Health Laboratory, National Centre for Infectious Diseas	National Public Health Laboratory, National Centre for Infectio	Mak TM, Octavia S, Cui L, Lin RTP
EPI_ISL_420099	hCoV-19/Singapore/22/2020	Asia / Singapore	2020-03-02	National Centre for Infectious Diseases	Programme in Emerging Infectious Diseases, Duke-NUS Med	Danielle E Anderson, Martin Linster, Yan Zhuang, Jayanthi Jayakumar, David CB Lye, Yee Sin Leo, Barnaby E Young, Yvonne CF Su, Gavin JD Smith
EPI_ISL_420100	hCoV-19/Singapore/23/2020	Asia / Singapore	2020-03-02	National Centre for Infectious Diseases	Programme in Emerging Infectious Diseases, Duke-NUS Med	Danielle E Anderson, Martin Linster, Yan Zhuang, Jayanthi Jayakumar, David CB Lye, Yee Sin Leo, Barnaby E Young, Yvonne CF Su, Gavin JD Smith
EPI_ISL_420101	hCoV-19/Singapore/24/2020	Asia / Singapore	2020-03-04	National Centre for Infectious Diseases	Programme in Emerging Infectious Diseases, Duke-NUS Med	Danielle E Anderson, Martin Linster, Yan Zhuang, Jayanthi Jayakumar, David CB Lye, Yee Sin Leo, Barnaby E Young, Yvonne CF Su, Gavin JD Smith
EPI_ISL_420102	hCoV-19/Singapore/25/2020	Asia / Singapore	2020-03-05	National Centre for Infectious Diseases	Programme in Emerging Infectious Diseases, Duke-NUS Med	Danielle E Anderson, Martin Linster, Yan Zhuang, Jayanthi Jayakumar, David CB Lye, Yee Sin Leo, Barnaby E Young, Yvonne CF Su, Gavin JD Smith
EPI_ISL_420103	hCoV-19/Singapore/26/2020	Asia / Singapore	2020-03-05	National Centre for Infectious Diseases	Programme in Emerging Infectious Diseases, Duke-NUS Med	Danielle E Anderson, Martin Linster, Yan Zhuang, Jayanthi Jayakumar, David CB Lye, Yee Sin Leo, Barnaby E Young, Yvonne CF Su, Gavin JD Smith
EPI_ISL_420104	hCoV-19/Singapore/27/2020	Asia / Singapore	2020-03-05	National Centre for Infectious Diseases	Programme in Emerging Infectious Diseases, Duke-NUS Med	Danielle E Anderson, Martin Linster, Yan Zhuang, Jayanthi Jayakumar, David CB Lye, Yee Sin Leo, Barnaby E Young, Yvonne CF Su, Gavin JD Smith
EPI_ISL_420105	hCoV-19/Singapore/28/2020	Asia / Singapore	2020-03-06	National Centre for Infectious Diseases	Programme in Emerging Infectious Diseases, Duke-NUS Med	Danielle E Anderson, Martin Linster, Yan Zhuang, Jayanthi Jayakumar, David CB Lye, Yee Sin Leo, Barnaby E Young, Yvonne CF Su, Gavin JD Smith
EPI_ISL_420106	hCoV-19/Singapore/29/2020	Asia / Singapore	2020-03-06	National Centre for Infectious Diseases	Programme in Emerging Infectious Diseases, Duke-NUS Med	Danielle E Anderson, Martin Linster, Yan Zhuang, Jayanthi Jayakumar, David CB Lye, Yee Sin Leo, Barnaby E Young, Yvonne CF Su, Gavin JD Smith
EPI_ISL_420107	hCoV-19/Singapore/30/2020	Asia / Singapore	2020-03-09	National Centre for Infectious Diseases	Programme in Emerging Infectious Diseases, Duke-NUS Med	Danielle E Anderson, Martin Linster, Yan Zhuang, Jayanthi Jayakumar, David CB Lye, Yee Sin Leo, Barnaby E Young, Yvonne CF Su, Gavin JD Smith
EPI_ISL_420108	hCoV-19/Singapore/31/2020	Asia / Singapore	2020-03-10	National Centre for Infectious Diseases	Programme in Emerging Infectious Diseases, Duke-NUS Med	Danielle E Anderson, Martin Linster, Yan Zhuang, Jayanthi Jayakumar, David CB Lye, Yee Sin Leo, Barnaby E Young, Yvonne CF Su, Gavin JD Smith
EPI_ISL_420109	hCoV-19/Singapore/32/2020	Asia / Singapore	2020-03-11	National Centre for Infectious Diseases	Programme in Emerging Infectious Diseases, Duke-NUS Med	Danielle E Anderson, Martin Linster, Yan Zhuang, Jayanthi Jayakumar, David CB Lye, Yee Sin Leo, Barnaby E Young, Yvonne CF Su, Gavin JD Smith
EPI_ISL_420110	hCoV-19/Singapore/33/2020	Asia / Singapore	2020-03-11	National Centre for Infectious Diseases	Programme in Emerging Infectious Diseases, Duke-NUS Med	Danielle E Anderson, Martin Linster, Yan Zhuang, Jayanthi Jayakumar, David CB Lye, Yee Sin Leo, Barnaby E Young, Yvonne CF Su, Gavin JD Smith
EPI_ISL_420111	hCoV-19/Singapore/34/2020	Asia / Singapore	2020-03-12	National Centre for Infectious Diseases	Programme in Emerging Infectious Diseases, Duke-NUS Med	Danielle E Anderson, Martin Linster, Yan Zhuang, Jayanthi Jayakumar, David CB Lye, Yee Sin Leo, Barnaby E Young, Yvonne CF Su, Gavin JD Smith
EPI_ISL_422428	hCoV-19/Singapore/35/2020	Asia / Singapore	2020-02-06	National Public Health Laboratory, National Centre for Infectious Diseas	National Public Health Laboratory, National Centre for Infectio	Mak TM, Octavia S, Cui L, Lin RTP
EPI_ISL_422429	hCoV-19/Singapore/36/2020	Asia / Singapore	2020-02-16	National Public Health Laboratory, National Centre for Infectious Diseas	National Public Health Laboratory, National Centre for Infectio	Mak TM, Octavia S, Cui L, Lin RTP
EPI_ISL_422430	hCoV-19/Singapore/37/2020	Asia / Singapore	2020-03-13	National Public Health Laboratory, National Centre for Infectious Diseas	National Public Health Laboratory, National Centre for Infectio	Mak TM, Octavia S, Cui L, Lin RTP
EPI_ISL_422431	hCoV-19/Singapore/38/2020	Asia / Singapore	2020-03-31	National Public Health Laboratory, National Centre for Infectious Diseas	National Public Health Laboratory, National Centre for Infectio	Mak TM, Octavia S, Cui L, Lin RTP
EPI_ISL_422432	hCoV-19/Singapore/39/2020	Asia / Singapore	2020-03-31	National Public Health Laboratory, National Centre for Infectious Diseas	National Public Health Laboratory, National Centre for Infectio	Mak TM, Octavia S, Cui L, Lin RTP
EPI_ISL_422433	hCoV-19/Singapore/40/2020	Asia / Singapore	2020-03-31	National Public Health Laboratory, National Centre for Infectious Diseas	National Public Health Laboratory, National Centre for Infectio	Mak TM, Octavia S, Cui L, Lin RTP
EPI_ISL_422434	hCoV-19/Singapore/41/2020	Asia / Singapore	2020-03-31	National Public Health Laboratory, National Centre for Infectious Diseas	National Public Health Laboratory, National Centre for Infectio	Mak TM, Octavia S, Cui L, Lin RTP
EPI_ISL_422435	hCoV-19/Singapore/42/2020	Asia / Singapore	2020-03-31	National Public Health Laboratory, National Centre for Infectious Diseas	National Public Health Laboratory, National Centre for Infectio	Mak TM, Octavia S, Cui L, Lin RTP
EPI_ISL_428822	hCoV-19/Singapore/43/2020	Asia / Singapore	2020-02-16	National Public Health Laboratory, National Centre for Infectious Diseas	National Public Health Laboratory, National Centre for Infectio	Mak TM, Octavia S, Chavatte JM, Cui L, Lin RTP
EPI_ISL_428823	hCoV-19/Singapore/44/2020	Asia / Singapore	2020-03-08	National Public Health Laboratory, National Centre for Infectious Diseas	National Public Health Laboratory, National Centre for Infectio	Mak TM, Octavia S, Chavatte JM, Cui L, Lin RTP
EPI_ISL_428824	hCoV-19/Singapore/45/2020	Asia / Singapore	2020-03-16	National Public Health Laboratory, National Centre for Infectious Diseas	National Public Health Laboratory, National Centre for Infectio	Mak TM, Octavia S, Chavatte JM, Cui L, Lin RTP
EPI_ISL_428825	hCoV-19/Singapore/46/2020	Asia / Singapore	2020-03-16	National Public Health Laboratory, National Centre for Infectious Diseas	National Public Health Laboratory, National Centre for Infectio	Mak TM, Octavia S, Chavatte JM, Cui L, Lin RTP
EPI_ISL_428826	hCoV-19/Singapore/47/2020	Asia / Singapore	2020-03-17	National Public Health Laboratory, National Centre for Infectious Diseas	National Public Health Laboratory, National Centre for Infectio	Mak TM, Octavia S, Chavatte JM, Cui L, Lin RTP
EPI_ISL_428827	hCoV-19/Singapore/48/2020	Asia / Singapore	2020-03-17	National Public Health Laboratory, National Centre for Infectious Diseas	National Public Health Laboratory, National Centre for Infectio	Mak TM, Octavia S, Chavatte JM, Cui L, Lin RTP
EPI_ISL_428828	hCoV-19/Singapore/49/2020	Asia / Singapore	2020-03-08	National Public Health Laboratory, National Centre for Infectious Diseas	National Public Health Laboratory, National Centre for Infectio	Mak TM, Octavia S, Chavatte JM, Cui L, Lin RTP
EPI_ISL_428829	hCoV-19/Singapore/50/2020	Asia / Singapore	2020-03-08	National Public Health Laboratory, National Centre for Infectious Diseas	National Public Health Laboratory, National Centre for Infectio	Mak TM, Octavia S, Chavatte JM, Cui L, Lin RTP
EPI_ISL_428830	hCoV-19/Singapore/51/2020	Asia / Singapore	2020-02-27	National Public Health Laboratory, National Centre for Infectious Diseas	National Public Health Laboratory, National Centre for Infectio	Mak TM, Octavia S, Chavatte JM, Cui L, Lin RTP
EPI_ISL_428831	hCoV-19/Singapore/52/2020	Asia / Singapore	2020-03-13	National Public Health Laboratory, National Centre for Infectious Diseas	National Public Health Laboratory, National Centre for Infectio	Mak TM, Octavia S, Chavatte JM, Cui L, Lin RTP
EPI_ISL_428832	hCoV-19/Singapore/53/2020	Asia / Singapore	2020-04-08	National Public Health Laboratory, National Centre for Infectious Diseas	National Public Health Laboratory, National Centre for Infectio	Mak TM, Octavia S, Chavatte JM, Cui L, Lin RTP
EPI_ISL_428833	hCoV-19/Singapore/54/2020	Asia / Singapore	2020-04-08	National Public Health Laboratory, National Centre for Infectious Diseas	National Public Health Laboratory, National Centre for Infectio	Mak TM, Octavia S, Chavatte JM, Cui L, Lin RTP
EPI_ISL_428834	hCoV-19/Singapore/55/2020	Asia / Singapore	2020-04-08	National Public Health Laboratory, National Centre for Infectious Diseas	National Public Health Laboratory, National Centre for Infectio	Mak TM, Octavia S, Chavatte JM, Cui L, Lin RTP
EPI_ISL_428835	hCoV-19/Singapore/56/2020	Asia / Singapore	2020-04-08	National Public Health Laboratory, National Centre for Infectious Diseas	National Public Health Laboratory, National Centre for Infectio	Mak TM, Octavia S, Chavatte JM, Cui L, Lin RTP
EPI_ISL_428836	hCoV-19/Singapore/57/2020	Asia / Singapore	2020-04-08	National Public Health Laboratory, National Centre for Infectious Diseas	National Public Health Laboratory, National Centre for Infectio	Mak TM, Octavia S, Chavatte JM, Cui L, Lin RTP
EPI_ISL_428837	hCoV-19/Singapore/58/2020	Asia / Singapore	2020-04-08	National Public Health Laboratory, National Centre for Infectious Diseas	National Public Health Laboratory, National Centre for Infectio	Mak TM, Octavia S, Chavatte JM, Cui L, Lin RTP
EPI_ISL_428838	hCoV-19/Singapore/59/2020	Asia / Singapore	2020-04-08	National Public Health Laboratory, National Centre for Infectious Diseas	National Public Health Laboratory, National Centre for Infectio	Mak TM, Octavia S, Chavatte JM, Cui L, Lin RTP
EPI_ISL_428839	hCoV-19/Singapore/60/2020	Asia / Singapore	2020-04-08	National Public Health Laboratory, National Centre for Infectious Diseas	National Public Health Laboratory, National Centre for Infectio	Mak TM, Octavia S, Chavatte JM, Cui L, Lin RTP
EPI_ISL_428840	hCoV-19/Singapore/61/2020	Asia / Singapore	2020-04-07	National Public Health Laboratory, National Centre for Infectious Diseas	National Public Health Laboratory, National Centre for Infectio	Mak TM, Octavia S, Chavatte JM, Cui L, Lin RTP
EPI_ISL_428841	hCoV-19/Singapore/62/2020	Asia / Singapore	2020-04-08	National Public Health Laboratory, National Centre for Infectious Diseas	National Public Health Laboratory, National Centre for Infectio	Mak TM, Octavia S, Chavatte JM, Cui L, Lin RTP
EPI_ISL_428842	hCoV-19/Singapore/63/2020	Asia / Singapore	2020-04-08	National Public Health Laboratory, National Centre for Infectious Diseas	National Public Health Laboratory, National Centre for Infectio	Mak TM, Octavia S, Chavatte JM, Cui L, Lin RTP
EPI_ISL_428843	hCoV-19/Singapore/64/2020	Asia / Singapore	2020-04-08	National Public Health Laboratory, National Centre for Infectious Diseas	National Public Health Laboratory, National Centre for Infectio	Mak TM, Octavia S, Chavatte JM, Cui L, Lin RTP
EPI_ISL_428844	hCoV-19/Singapore/65/2020	Asia / Singapore	2020-04-08	National Public Health Laboratory, National Centre for Infectious Diseas	National Public Health Laboratory, National Centre for Infectio	Mak TM, Octavia S, Chavatte JM, Cui L, Lin RTP
EPI_ISL_428845	hCoV-19/Singapore/66/2020	Asia / Singapore	2020-04-08	National Public Health Laboratory, National Centre for Infectious Diseas	National Public Health Laboratory, National Centre for Infectio	Mak TM, Octavia S, Chavatte JM, Cui L, Lin RTP
EPI_ISL_428846	hCoV-19/Singapore/67/2020	Asia / Singapore	2020-04-15	National Public Health Laboratory, National Centre for Infectious Diseas	National Public Health Laboratory, National Centre for Infectio	Mak TM, Octavia S, Chavatte JM, Cui L, Lin RTP
EPI_ISL_428847	hCoV-19/Singapore/68/2020	Asia / Singapore	2020-04-15	National Public Health Laboratory, National Centre for Infectious Diseas	National Public Health Laboratory, National Centre for Infectio	Mak TM, Octavia S, Chavatte JM, Cui L, Lin RTP
EPI_ISL_428848	hCoV-19/Singapore/69/2020	Asia / Singapore	2020-04-15	National Public Health Laboratory, National Centre for Infectious Diseas	National Public Health Laboratory, National Centre for Infectio	Mak TM, Octavia S, Chavatte JM, Cui L, Lin RTP
EPI_ISL_428849	hCoV-19/Singapore/70/2020	Asia / Singapore	2020-04-15	National Public Health Laboratory, National Centre for Infectious Diseas	National Public Health Laboratory, National Centre for Infectio	Mak TM, Octavia S, Chavatte JM, Cui L, Lin RTP
EPI_ISL_428850	hCoV-19/Singapore/71/2020	Asia / Singapore	2020-04-15	National Public Health Laboratory, National Centre for Infectious Diseas	National Public Health Laboratory, National Centre for Infectio	Mak TM, Octavia S, Chavatte JM, Cui L, Lin RTP
EPI_ISL_403962	hCoV-19/Nonthaburi/61/2020	Asia / Thailand / Nonthaburi	2020-01-08	Bamrasnaradura Hospital	1. Department of Medical Sciences, Ministry of Public Health,	Pilailuk,Okada; Siripaporn,Phuygun; Thanutsapa,Thanadachakul; Supaporn,Wacharapluesadee; Sittiporn,Parnmen; Warawan,Wongboot; Sunthareeya,Waicharoen; Rome,Buathong; Malinee,Chittaganpitch; Nanthawan,Mekha
EPI_ISL_403963	hCoV-19/Nonthaburi/74/2020	Asia / Thailand / Nonthaburi	2020-01-13	Bamrasnaradura Hospital	1. Department of Medical Sciences, Ministry of Public Health,	Pilailuk,Okada; Siripaporn,Phuygun; Thanutsapa,Thanadachakul; Supaporn,Wacharapluesadee; Sittiporn,Parnmen; Warawan,Wongboot; Sunthareeya,Waicharoen; Rome,Buathong; Malinee,Chittaganpitch; Nanthawan,Mekha
EPI_ISL_423039	hCoV-19/Thailand/Bangkok-0017/2020	Asia / Thailand / Bangkok	2020-03-21	Ramathibodi Hospital	COVID-19 Network Investigations (CONI) Alliance	Elizabeth Batty, Wasun Chantratita, Thanat Chookajorn, Stefan Fernandez, Angkana Huang, Anthony R. Jones, Khajohn Joonsalak, Chonticha Klungtong, Theerarat Kochakarn, Namfon Kotanan, Krittikorn Kumpornsin, Wudtichai Manasatienkij, Bhakbhoom Panthan, Ekawat Pasomsub, Insee Sensorn, Arporn Wangwiwatsin
EPI_ISL_423040	hCoV-19/Thailand/Bangkok-0019/2020	Asia / Thailand / Bangkok	2020-03-20	Ramathibodi Hospital	COVID-19 Network Investigations (CONI) Alliance	Elizabeth Batty, Wasun Chantratita, Thanat Chookajorn, Stefan Fernandez, Angkana Huang, Anthony R. Jones, Khajohn Joonsalak, Chonticha Klungtong, Theerarat Kochakarn, Namfon Kotanan, Krittikorn Kumpornsin, Wudtichai Manasatienkij, Bhakbhoom Panthan, Ekawat Pasomsub, Insee Sensorn, Arporn Wangwiwatsin
EPI_ISL_423041	hCoV-19/Thailand/Bangkok-0020/2020	Asia / Thailand / Bangkok	2020-03-21	Ramathibodi Hospital	COVID-19 Network Investigations (CONI) Alliance	Elizabeth Batty, Wasun Chantratita, Thanat Chookajorn, Stefan Fernandez, Angkana Huang, Anthony R. Jones, Khajohn Joonsalak, Chonticha Klungtong, Theerarat Kochakarn, Namfon Kotanan, Krittikorn Kumpornsin, Wudtichai Manasatienkij, Bhakbhoom Panthan, Ekawat Pasomsub, Insee Sensorn, Arporn Wangwiwatsin
EPI_ISL_423042	hCoV-19/Thailand/Bangkok-0021/2020	Asia / Thailand / Bangkok	2020-03-24	Ramathibodi Hospital	COVID-19 Network Investigations (CONI) Alliance	Elizabeth Batty, Wasun Chantratita, Thanat Chookajorn, Stefan Fernandez, Angkana Huang, Anthony R. Jones, Khajohn Joonsalak, Chonticha Klungtong, Theerarat Kochakarn, Namfon Kotanan, Krittikorn Kumpornsin, Wudtichai Manasatienkij, Bhakbhoom Panthan, Ekawat Pasomsub, Insee Sensorn, Arporn Wangwiwatsin
EPI_ISL_423043	hCoV-19/Thailand/Bangkok-0022/2020	Asia / Thailand / Bangkok	2020-03-18	Ramathibodi Hospital	COVID-19 Network Investigations (CONI) Alliance	Elizabeth Batty, Wasun Chantratita, Thanat Chookajorn, Stefan Fernandez, Angkana Huang, Anthony R. Jones, Khajohn Joonsalak, Chonticha Klungtong, Theerarat Kochakarn, Namfon Kotanan, Krittikorn Kumpornsin, Wudtichai Manasatienkij, Bhakbhoom Panthan, Ekawat Pasomsub, Insee Sensorn, Arporn Wangwiwatsin
EPI_ISL_429164	hCoV-19/Thailand/Bangkok-0025/2020	Asia / Thailand / Bangkok	2020-03-13	Ramathibodi Hospital	COVID-19 Network Investigations (CONI) Alliance	Elizabeth Batty, Wasun Chantratita, Thanat Chookajorn, Stefan Fernandez, Angkana Huang, Poramate Jiaranai, Anthony R. Jones, Khajohn Joonsalak, Chonticha Klungtong, Theerarat Kochakarn, Namfon Kotanan, Krittikorn Kumpornsin, Wudtichai Manasatienkij, Bhakbhoom Panthan, Ekawat Pasomsub, Kingkan Rakmanee, Insee Sensorn, Janjira Thaipadungpanit, Arporn Wangwiwatsin,Treewat Watthanachockchai
EPI_ISL_429165	hCoV-19/Thailand/Bangkok-0026/2020	Asia / Thailand / Bangkok	2020-03-13	Ramathibodi Hospital	COVID-19 Network Investigations (CONI) Alliance	Elizabeth Batty, Wasun Chantratita, Thanat Chookajorn, Stefan Fernandez, Angkana Huang, Poramate Jiaranai, Anthony R. Jones, Khajohn Joonsalak, Chonticha Klungtong, Theerarat Kochakarn, Namfon Kotanan, Krittikorn Kumpornsin, Wudtichai Manasatienkij, Bhakbhoom Panthan, Ekawat Pasomsub, Kingkan Rakmanee, Insee Sensorn, Janjira Thaipadungpanit, Arporn Wangwiwatsin,Treewat Watthanachockchai
EPI_ISL_429166	hCoV-19/Thailand/Bangkok-0028/2020	Asia / Thailand / Bangkok	2020-03-13	Ramathibodi Hospital	COVID-19 Network Investigations (CONI) Alliance	Elizabeth Batty, Wasun Chantratita, Thanat Chookajorn, Stefan Fernandez, Angkana Huang, Poramate Jiaranai, Anthony R. Jones, Khajohn Joonsalak, Chonticha Klungtong, Theerarat Kochakarn, Namfon Kotanan, Krittikorn Kumpornsin, Wudtichai Manasatienkij, Bhakbhoom Panthan, Ekawat Pasomsub, Kingkan Rakmanee, Insee Sensorn, Janjira Thaipadungpanit, Arporn Wangwiwatsin,Treewat Watthanachockchai
EPI_ISL_429167	hCoV-19/Thailand/Bangkok-0029/2020	Asia / Thailand / Bangkok	2020-03-13	Ramathibodi Hospital	COVID-19 Network Investigations (CONI) Alliance	Elizabeth Batty, Wasun Chantratita, Thanat Chookajorn, Stefan Fernandez, Angkana Huang, Poramate Jiaranai, Anthony R. Jones, Khajohn Joonsalak, Chonticha Klungtong, Theerarat Kochakarn, Namfon Kotanan, Krittikorn Kumpornsin, Wudtichai Manasatienkij, Bhakbhoom Panthan, Ekawat Pasomsub, Kingkan Rakmanee, Insee Sensorn, Janjira Thaipadungpanit, Arporn Wangwiwatsin,Treewat Watthanachockchai
EPI_ISL_429168	hCoV-19/Thailand/Bangkok-0030/2020	Asia / Thailand / Bangkok	2020-03-17	Ramathibodi Hospital	COVID-19 Network Investigations (CONI) Alliance	Elizabeth Batty, Wasun Chantratita, Thanat Chookajorn, Stefan Fernandez, Angkana Huang, Poramate Jiaranai, Anthony R. Jones, Khajohn Joonsalak, Chonticha Klungtong, Theerarat Kochakarn, Namfon Kotanan, Krittikorn Kumpornsin, Wudtichai Manasatienkij, Bhakbhoom Panthan, Ekawat Pasomsub, Kingkan Rakmanee, Insee Sensorn, Janjira Thaipadungpanit, Arporn Wangwiwatsin,Treewat Watthanachockchai
EPI_ISL_429169	hCoV-19/Thailand/Bangkok-0033/2020	Asia / Thailand / Bangkok	2020-03-17	Ramathibodi Hospital	COVID-19 Network Investigations (CONI) Alliance	Elizabeth Batty, Wasun Chantratita, Thanat Chookajorn, Stefan Fernandez, Angkana Huang, Poramate Jiaranai, Anthony R. Jones, Khajohn Joonsalak, Chonticha Klungtong, Theerarat Kochakarn, Namfon Kotanan, Krittikorn Kumpornsin, Wudtichai Manasatienkij, Bhakbhoom Panthan, Ekawat Pasomsub, Kingkan Rakmanee, Insee Sensorn, Janjira Thaipadungpanit, Arporn Wangwiwatsin,Treewat Watthanachockchai
EPI_ISL_429170	hCoV-19/Thailand/Bangkok-0034/2020	Asia / Thailand / Bangkok	2020-03-17	Ramathibodi Hospital	COVID-19 Network Investigations (CONI) Alliance	Elizabeth Batty, Wasun Chantratita, Thanat Chookajorn, Stefan Fernandez, Angkana Huang, Poramate Jiaranai, Anthony R. Jones, Khajohn Joonsalak, Chonticha Klungtong, Theerarat Kochakarn, Namfon Kotanan, Krittikorn Kumpornsin, Wudtichai Manasatienkij, Bhakbhoom Panthan, Ekawat Pasomsub, Kingkan Rakmanee, Insee Sensorn, Janjira Thaipadungpanit, Arporn Wangwiwatsin,Treewat Watthanachockchai
EPI_ISL_429171	hCoV-19/Thailand/Bangkok-0035/2020	Asia / Thailand / Bangkok	2020-03-18	Ramathibodi Hospital	COVID-19 Network Investigations (CONI) Alliance	Elizabeth Batty, Wasun Chantratita, Thanat Chookajorn, Stefan Fernandez, Angkana Huang, Poramate Jiaranai, Anthony R. Jones, Khajohn Joonsalak, Chonticha Klungtong, Theerarat Kochakarn, Namfon Kotanan, Krittikorn Kumpornsin, Wudtichai Manasatienkij, Bhakbhoom Panthan, Ekawat Pasomsub, Kingkan Rakmanee, Insee Sensorn, Janjira Thaipadungpanit, Arporn Wangwiwatsin,Treewat Watthanachockchai
EPI_ISL_429172	hCoV-19/Thailand/Bangkok-0036/2020	Asia / Thailand / Bangkok	2020-03-21	Ramathibodi Hospital	COVID-19 Network Investigations (CONI) Alliance	Elizabeth Batty, Wasun Chantratita, Thanat Chookajorn, Stefan Fernandez, Angkana Huang, Poramate Jiaranai, Anthony R. Jones, Khajohn Joonsalak, Chonticha Klungtong, Theerarat Kochakarn, Namfon Kotanan, Krittikorn Kumpornsin, Wudtichai Manasatienkij, Bhakbhoom Panthan, Ekawat Pasomsub, Kingkan Rakmanee, Insee Sensorn, Janjira Thaipadungpanit, Arporn Wangwiwatsin,Treewat Watthanachockchai
EPI_ISL_429173	hCoV-19/Thailand/Bangkok-0037/2020	Asia / Thailand / Bangkok	2020-03-21	Ramathibodi Hospital	COVID-19 Network Investigations (CONI) Alliance	Elizabeth Batty, Wasun Chantratita, Thanat Chookajorn, Stefan Fernandez, Angkana Huang, Poramate Jiaranai, Anthony R. Jones, Khajohn Joonsalak, Chonticha Klungtong, Theerarat Kochakarn, Namfon Kotanan, Krittikorn Kumpornsin, Wudtichai Manasatienkij, Bhakbhoom Panthan, Ekawat Pasomsub, Kingkan Rakmanee, Insee Sensorn, Janjira Thaipadungpanit, Arporn Wangwiwatsin,Treewat Watthanachockchai
EPI_ISL_429174	hCoV-19/Thailand/Bangkok-0038/2020	Asia / Thailand / Bangkok	2020-03-25	Ramathibodi Hospital	COVID-19 Network Investigations (CONI) Alliance	Elizabeth Batty, Wasun Chantratita, Thanat Chookajorn, Stefan Fernandez, Angkana Huang, Poramate Jiaranai, Anthony R. Jones, Khajohn Joonsalak, Chonticha Klungtong, Theerarat Kochakarn, Namfon Kotanan, Krittikorn Kumpornsin, Wudtichai Manasatienkij, Bhakbhoom Panthan, Ekawat Pasomsub, Kingkan Rakmanee, Insee Sensorn, Janjira Thaipadungpanit, Arporn Wangwiwatsin,Treewat Watthanachockchai
EPI_ISL_429175	hCoV-19/Thailand/Bangkok-0039/2020	Asia / Thailand / Bangkok	2020-03-28	Ramathibodi Hospital	COVID-19 Network Investigations (CONI) Alliance	Elizabeth Batty, Wasun Chantratita, Thanat Chookajorn, Stefan Fernandez, Angkana Huang, Poramate Jiaranai, Anthony R. Jones, Khajohn Joonsalak, Chonticha Klungtong, Theerarat Kochakarn, Namfon Kotanan, Krittikorn Kumpornsin, Wudtichai Manasatienkij, Bhakbhoom Panthan, Ekawat Pasomsub, Kingkan Rakmanee, Insee Sensorn, Janjira Thaipadungpanit, Arporn Wangwiwatsin,Treewat Watthanachockchai
EPI_ISL_429176	hCoV-19/Thailand/Bangkok-0040/2020	Asia / Thailand / Bangkok	2020-03-28	Ramathibodi Hospital	COVID-19 Network Investigations (CONI) Alliance	Elizabeth Batty, Wasun Chantratita, Thanat Chookajorn, Stefan Fernandez, Angkana Huang, Poramate Jiaranai, Anthony R. Jones, Khajohn Joonsalak, Chonticha Klungtong, Theerarat Kochakarn, Namfon Kotanan, Krittikorn Kumpornsin, Wudtichai Manasatienkij, Bhakbhoom Panthan, Ekawat Pasomsub, Kingkan Rakmanee, Insee Sensorn, Janjira Thaipadungpanit, Arporn Wangwiwatsin,Treewat Watthanachockchai
EPI_ISL_429177	hCoV-19/Thailand/Bangkok-0041/2020	Asia / Thailand / Bangkok	2020-03-28	Ramathibodi Hospital	COVID-19 Network Investigations (CONI) Alliance	Elizabeth Batty, Wasun Chantratita, Thanat Chookajorn, Stefan Fernandez, Angkana Huang, Poramate Jiaranai, Anthony R. Jones, Khajohn Joonsalak, Chonticha Klungtong, Theerarat Kochakarn, Namfon Kotanan, Krittikorn Kumpornsin, Wudtichai Manasatienkij, Bhakbhoom Panthan, Ekawat Pasomsub, Kingkan Rakmanee, Insee Sensorn, Janjira Thaipadungpanit, Arporn Wangwiwatsin,Treewat Watthanachockchai
EPI_ISL_429178	hCoV-19/Thailand/Bangkok-0042/2020	Asia / Thailand / Bangkok	2020-03-28	Ramathibodi Hospital	COVID-19 Network Investigations (CONI) Alliance	Elizabeth Batty, Wasun Chantratita, Thanat Chookajorn, Stefan Fernandez, Angkana Huang, Poramate Jiaranai, Anthony R. Jones, Khajohn Joonsalak, Chonticha Klungtong, Theerarat Kochakarn, Namfon Kotanan, Krittikorn Kumpornsin, Wudtichai Manasatienkij, Bhakbhoom Panthan, Ekawat Pasomsub, Kingkan Rakmanee, Insee Sensorn, Janjira Thaipadungpanit, Arporn Wangwiwatsin,Treewat Watthanachockchai
EPI_ISL_429179	hCoV-19/Thailand/Bangkok-0043/2020	Asia / Thailand / Bangkok	2020-03-28	Ramathibodi Hospital	COVID-19 Network Investigations (CONI) Alliance	Elizabeth Batty, Wasun Chantratita, Thanat Chookajorn, Stefan Fernandez, Angkana Huang, Poramate Jiaranai, Anthony R. Jones, Khajohn Joonsalak, Chonticha Klungtong, Theerarat Kochakarn, Namfon Kotanan, Krittikorn Kumpornsin, Wudtichai Manasatienkij, Bhakbhoom Panthan, Ekawat Pasomsub, Kingkan Rakmanee, Insee Sensorn, Janjira Thaipadungpanit, Arporn Wangwiwatsin,Treewat Watthanachockchai
EPI_ISL_429180	hCoV-19/Thailand/Bangkok-0044/2020	Asia / Thailand / Bangkok	2020-03-28	Ramathibodi Hospital	COVID-19 Network Investigations (CONI) Alliance	Elizabeth Batty, Wasun Chantratita, Thanat Chookajorn, Stefan Fernandez, Angkana Huang, Poramate Jiaranai, Anthony R. Jones, Khajohn Joonsalak, Chonticha Klungtong, Theerarat Kochakarn, Namfon Kotanan, Krittikorn Kumpornsin, Wudtichai Manasatienkij, Bhakbhoom Panthan, Ekawat Pasomsub, Kingkan Rakmanee, Insee Sensorn, Janjira Thaipadungpanit, Arporn Wangwiwatsin,Treewat Watthanachockchai
EPI_ISL_429181	hCoV-19/Thailand/Bangkok-0045/2020	Asia / Thailand / Bangkok	2020-03-28	Ramathibodi Hospital	COVID-19 Network Investigations (CONI) Alliance	Elizabeth Batty, Wasun Chantratita, Thanat Chookajorn, Stefan Fernandez, Angkana Huang, Poramate Jiaranai, Anthony R. Jones, Khajohn Joonsalak, Chonticha Klungtong, Theerarat Kochakarn, Namfon Kotanan, Krittikorn Kumpornsin, Wudtichai Manasatienkij, Bhakbhoom Panthan, Ekawat Pasomsub, Kingkan Rakmanee, Insee Sensorn, Janjira Thaipadungpanit, Arporn Wangwiwatsin,Treewat Watthanachockchai
EPI_ISL_429182	hCoV-19/Thailand/Bangkok-0046/2020	Asia / Thailand / Bangkok	2020-03-28	Ramathibodi Hospital	COVID-19 Network Investigations (CONI) Alliance	Elizabeth Batty, Wasun Chantratita, Thanat Chookajorn, Stefan Fernandez, Angkana Huang, Poramate Jiaranai, Anthony R. Jones, Khajohn Joonsalak, Chonticha Klungtong, Theerarat Kochakarn, Namfon Kotanan, Krittikorn Kumpornsin, Wudtichai Manasatienkij, Bhakbhoom Panthan, Ekawat Pasomsub, Kingkan Rakmanee, Insee Sensorn, Janjira Thaipadungpanit, Arporn Wangwiwatsin,Treewat Watthanachockchai
EPI_ISL_429183	hCoV-19/Thailand/Bangkok-0047/2020	Asia / Thailand / Bangkok	2020-03-28	Ramathibodi Hospital	COVID-19 Network Investigations (CONI) Alliance	Elizabeth Batty, Wasun Chantratita, Thanat Chookajorn, Stefan Fernandez, Angkana Huang, Poramate Jiaranai, Anthony R. Jones, Khajohn Joonsalak, Chonticha Klungtong, Theerarat Kochakarn, Namfon Kotanan, Krittikorn Kumpornsin, Wudtichai Manasatienkij, Bhakbhoom Panthan, Ekawat Pasomsub, Kingkan Rakmanee, Insee Sensorn, Janjira Thaipadungpanit, Arporn Wangwiwatsin,Treewat Watthanachockchai
EPI_ISL_429184	hCoV-19/Thailand/Bangkok-0048/2020	Asia / Thailand / Bangkok	2020-03-28	Ramathibodi Hospital	COVID-19 Network Investigations (CONI) Alliance	Elizabeth Batty, Wasun Chantratita, Thanat Chookajorn, Stefan Fernandez, Angkana Huang, Poramate Jiaranai, Anthony R. Jones, Khajohn Joonsalak, Chonticha Klungtong, Theerarat Kochakarn, Namfon Kotanan, Krittikorn Kumpornsin, Wudtichai Manasatienkij, Bhakbhoom Panthan, Ekawat Pasomsub, Kingkan Rakmanee, Insee Sensorn, Janjira Thaipadungpanit, Arporn Wangwiwatsin,Treewat Watthanachockchai
EPI_ISL_430837	hCoV-19/Thailand/TNIC-1889/2020	Asia / Thailand / Nonthaburi	2020-03-03	n/a	Thai National Influenza Center, Department of medical Scienc	Pilailuk,Okada; Siripaporn,Phuygun; Thanutsapa,Thanadachakul;Sittiporn,Parnmen;Warawan,Wongboot;Sunthareeya,Waicharoen; Malinee,Chittaganpitch
EPI_ISL_430841	hCoV-19/Thailand/NIH_2492/2020	Asia / Thailand / Bangkok	2020-03-17	Praram 9 Hospital	National Institute of Health. Department of medical Sciences,	Pilailuk,Okada; Siripaporn,Phuygun; Thanutsapa,Thanadachakul; Sittiporn,Parnmen;Warawan,Wongboot; Sunthareeya,Waicharoen; Malinee,Chittaganpitch
EPI_ISL_430842	hCoV-19/Thailand/NIH-2982/2020	Asia / Thailand / Nonthaburi	2020-03-18	Central chest Institute of Thailand	National Institute of Health. Department of medical Sciences,	Pilailuk,Okada; Siripaporn,Phuygun; Thanutsapa,Thanadachakul; Sittiporn,Parnmen;Warawan,Wongboot; Sunthareeya,Waicharoen; Malinee,Chittaganpitch
EPI_ISL_408668	hCoV-19/Vietnam/VR03-38142/2020	Asia / Vietnam / Thanh Hoa	2020-01-24	National Influenza Center - National Institute of Hygiene and Epidemio	National Influenza Center - National Institute of Hygiene and E	Ung Thi Hong Trang, Hoang Vu Mai Phuong, Nguyen Le Khanh Hang, Nguyen Vu Son, Le Thi Thanh, Vuong Duc Cuong, Nguyen Phuong Anh, Pham Thi Hien, Tran Thu Huong, Le Thi Quynh Mai,
EPI_ISL_416427	hCoV-19/Vietnam/38142/2020	Asia / Vietnam / Thanhhoa	2020-01-24	National Influenza Center, National Institute of Hygiene and Epidemiolo	National Influenza Center, National Institute of Hygiene and E	Le Quynh Mai, Taichiro Takemura, Meng Ling Moi, Takeshi Nabeshima, Nguyen Le Khanh Hang, Hoang Vu Mai Phuong, Ung Thi Hong Trang, Le Thi Thanh, Nguyen Vu Son, Vuong Duc Cuong, Pham Thi Hien, Tran Thu Huong, Nguyen Phuong Anh, Pham Hong Quynh Anh, Kouichi Morita, Futoshi Hasebe, Dang Duc Anh
EPI_ISL_416428	hCoV-19/Vietnam/39607/2020	Asia / Vietnam / Quangning	2020-03-07	National Influenza Center, National Institute of Hygiene and Epidemiolo	National Influenza Center, National Institute of Hygiene and E	Le Quynh Mai, Taichiro Takemura, Meng Ling Moi, Takeshi Nabeshima, Nguyen Le Khanh Hang, Hoang Vu Mai Phuong, Ung Thi Hong Trang, Le Thi Thanh, Nguyen Vu Son, Vuong Duc Cuong, Pham Thi Hien, Tran Thu Huong, Nguyen Phuong Anh, Pham Hong Quynh Anh, Kouichi Morita, Futoshi Hasebe, Dang Duc Anh
EPI_ISL_416429	hCoV-19/Vietnam/CM99/2020	Asia / Vietnam / Vinhphuc	2020-02-11	National Influenza Center, National Institute of Hygiene and Epidemiolo	National Influenza Center, National Institute of Hygiene and E	Le Quynh Mai, Taichiro Takemura, Meng Ling Moi, Takeshi Nabeshima, Nguyen Le Khanh Hang, Hoang Vu Mai Phuong, Ung Thi Hong Trang, Le Thi Thanh, Nguyen Vu Son, Vuong Duc Cuong, Pham Thi Hien, Tran Thu Huong, Nguyen Phuong Anh, Pham Hong Quynh Anh, Kouichi Morita, Futoshi Hasebe, Dang Duc Anh
EPI_ISL_416430	hCoV-19/Vietnam/CM295/2020	Asia / Vietnam / Hanoi	2020-03-06	National Influenza Center, National Institute of Hygiene and Epidemiolo	National Influenza Center, National Institute of Hygiene and E	Le Quynh Mai, Taichiro Takemura, Meng Ling Moi, Takeshi Nabeshima, Nguyen Le Khanh Hang, Hoang Vu Mai Phuong, Ung Thi Hong Trang, Le Thi Thanh, Nguyen Vu Son, Vuong Duc Cuong, Pham Thi Hien, Tran Thu Huong, Nguyen Phuong Anh, Pham Hong Quynh Anh, Kouichi Morita, Futoshi Hasebe, Dang Duc Anh
EPI_ISL_416431	hCoV-19/Vietnam/CM296/2020	Asia / Vietnam / Hanoi	2020-03-06	National Influenza Center, National Institute of Hygiene and Epidemiolo	National Influenza Center, National Institute of Hygiene and E	Le Quynh Mai, Taichiro Takemura, Meng Ling Moi, Takeshi Nabeshima, Nguyen Le Khanh Hang, Hoang Vu Mai Phuong, Ung Thi Hong Trang, Le Thi Thanh, Nguyen Vu Son, Vuong Duc Cuong, Pham Thi Hien, Tran Thu Huong, Nguyen Phuong Anh, Pham Hong Quynh Anh, Kouichi Morita, Futoshi Hasebe, Dang Duc Anh
EPI_ISL_418267	hCoV-19/Vietnam/19-02S/2020	Asia / Vietnam / Ho Chi Minh City	2020-01-22	unknown	Microbiology and Immunology department	Nguyen,H.T., Cao,T.M., Pham,H.T.T., Vu,N.P.H., Dao,M.H., Huynh,L.T.K., Nguyen,L.T., Nguyen,N.T., Nguyen,T.T.N., Nguyen,A.H., Luong,Q.C., Nguyen,T.V., Tran,K.C., Pham,Q.D., Tran,T., Hoang,C.Q., Nguyen,T.T., Le,H.Q., Phung,T.M., Vo,T.N.A., Nguyen,S.N., Pham,D.T., Nguyen,T.V. and Phan,L.T.
EPI_ISL_418269	hCoV-19/Vietnam/19-01S/2020	Asia / Vietnam / Ho Chi Minh City	2020-01-22	unknown	Microbiology and Immunology department	Cao,T.M., Nguyen,H.T., Pham,H.T.T., Vu,N.P.H., Dao,M.H., Huynh,L.T.K., Nguyen,L.T., Nguyen,N.T., Nguyen,T.T.N., Nguyen,A.H., Luong,Q.C., Nguyen,T.V., Tran,K.C., Pham,Q.D., Tran,T., Hoang,C.Q., Nguyen,T.T., Le,H.Q., Phung,T.M., Vo,T.N.A., Nguyen,S.N., Pham,D.T., Phan,L.T. and Nguyen,T.V.

Supplementary Data 2: Strains' date of collection with regards to the country of origin's date of limiting border entry

Source: https://www.nytimes.com/article/coronavirus-travel-restrictions.html

**Country Name**	**Date border control started**	**Strain collected before border control**	**Strains collected after border control**
Cambodia	2020-03-31	Cambodia/0012/2020|EPI_ISL_411902|2020-01-27	
Malaysia	2020-03-16	Malaysia/186197/2020|EPI_ISL_417919|2020-03-14<br>Malaysia/IMR_WC085/2020|EPI_ISL_430443|2020-01-28<br>Malaysia/IMR_WC1097/2020|EPI_ISL_430441|2020-02-29<br>Malaysia/IMR_WC1098/2020|EPI_ISL_430442|2020-02-29<br>Malaysia/IMR_WC1170/2020|EPI_ISL_430440|2020-03-05<br>Malaysia/IMR_WC1177/2020|EPI_ISL_430439|2020-03-05<br>Malaysia/IMR_WC627/2020|EPI_ISL_430444|2020-02-12<br>Malaysia/MKAK-CL-2020-5045/2020|EPI_ISL_416829|2020-02-20<br>Malaysia/MKAK-CL-2020-5047/2020|EPI_ISL_416866|2020-02-20<br>Malaysia/MKAK-CL-2020-5049/2020|EPI_ISL_416884|2020-02-20<br>Malaysia/MKAK-CL-2020-5096/2020|EPI_ISL_416885|2020-02-20<br>Malaysia/MKAK-CL-2020-6430/2020|EPI_ISL_416886|2020-02-20<br>Malaysia/MKAK-CL-2020-7554/2020|EPI_ISL_416907|2020-02-20	Malaysia/188407/2020|EPI_ISL_417918|2020-03-18<br>Malaysia/189332/2020|EPI_ISL_417917|2020-03-20<br>Malaysia/190300/2020|EPI_ISL_417920|2020-03-22
Philippines	2020-03-14	Philippines/RITM-01/2020|EPI_ISL_430838|2020-03-10<br>Philippines/RITM-02/2020|EPI_ISL_430839|2020-03-11<br>Philippines/RITM-04/2020|EPI_ISL_430840|2020-03-08	Philippines/PGC001/2020|EPI_ISL_431833|2020-03-22<br>Philippines/PGC002/2020|EPI_ISL_434554|2020-03-26<br>Philippines/PGC003/2020|EPI_ISL_434555|2020-03-26<br>Philippines/PGC005/2020|EPI_ISL_434557|2020-03-27<br>Philippines/PGC006/2020|EPI_ISL_434558|2020-03-28<br>Philippines/RITM-05/2020|EPI_ISL_430843|2020-03-22<br>Philippines/RITM-06/2020|EPI_ISL_430844|2020-03-22<br>Philippines/RITM-07/2020|EPI_ISL_430845|2020-03-23<br>Philippines/RITM-03/2020|EPI_ISL_430456|2020-03-21
Singapore	2020-03-23	Singapore/1/2020|EPI_ISL_406973|2020-01-23<br>Singapore/10/2020|EPI_ISL_410716|2020-02-04<br>Singapore/11/2020|EPI_ISL_410719|2020-02-02<br>Singapore/12/2020|EPI_ISL_414378|2020-02-17<br>Singapore/12Clin/2020|EPI_ISL_418995|2020-02-28<br>Singapore/13/2020|EPI_ISL_414379|2020-02-18<br>Singapore/14/2020|EPI_ISL_414380|2020-02-13<br>Singapore/14Clin/2020|EPI_ISL_418998|2020-02-14<br>Singapore/15/2020|EPI_ISL_418996|2020-01-27<br>Singapore/16/2020|EPI_ISL_418997|2020-02-06<br>Singapore/17/2020|EPI_ISL_418992|2020-02-10<br>Singapore/18/2020|EPI_ISL_418999|2020-03-01<br>Singapore/19/2020|EPI_ISL_419001|2020-03-02<br>Singapore/2/2020|EPI_ISL_407987|2020-01-25<br>Singapore/20/2020|EPI_ISL_418993|2020-01-29<br>Singapore/21/2020|EPI_ISL_419000|2020-02-13<br>Singapore/22/2020|EPI_ISL_420099|2020-03-02<br>Singapore/23/2020|EPI_ISL_420100|2020-03-02<br>Singapore/24/2020|EPI_ISL_420101|2020-03-04<br>Singapore/25/2020|EPI_ISL_420102|2020-03-05<br>Singapore/26/2020|EPI_ISL_420103|2020-03-05<br>Singapore/27/2020|EPI_ISL_420104|2020-03-05<br>Singapore/28/2020|EPI_ISL_420105|2020-03-06<br>Singapore/29/2020|EPI_ISL_420106|2020-03-06<br>Singapore/3/2020|EPI_ISL_407988|2020-02-01<br>Singapore/30/2020|EPI_ISL_420107|2020-03-09<br>Singapore/31/2020|EPI_ISL_420108|2020-03-10<br>Singapore/32/2020|EPI_ISL_420109|2020-03-11<br>Singapore/33/2020|EPI_ISL_420110|2020-03-11<br>Singapore/34/2020|EPI_ISL_420111|2020-03-12<br>Singapore/35/2020|EPI_ISL_422428|2020-02-06<br>Singapore/36/2020|EPI_ISL_422429|2020-02-16<br>Singapore/37/2020|EPI_ISL_422430|2020-03-13<br>Singapore/3Clin/2020|EPI_ISL_418994|2020-01-29<br>Singapore/4/2020|EPI_ISL_410535|2020-02-03<br>Singapore/43/2020|EPI_ISL_428822|2020-02-16<br>Singapore/44/2020|EPI_ISL_428823|2020-03-08<br>Singapore/45/2020|EPI_ISL_428824|2020-03-16<br>Singapore/46/2020|EPI_ISL_428825|2020-03-16<br>Singapore/47/2020|EPI_ISL_428826|2020-03-17<br>Singapore/48/2020|EPI_ISL_428827|2020-03-17<br>Singapore/49/2020|EPI_ISL_428828|2020-03-08<br>Singapore/5/2020|EPI_ISL_410536|2020-02-06<br>Singapore/50/2020|EPI_ISL_428829|2020-03-08<br>Singapore/51/2020|EPI_ISL_428830|2020-02-27<br>Singapore/52/2020|EPI_ISL_428831|2020-03-13<br>Singapore/6/2020|EPI_ISL_410537|2020-02-09<br>Singapore/7/2020|EPI_ISL_410713|2020-01-27<br>Singapore/8/2020|EPI_ISL_410714|2020-02-03<br>Singapore/9/2020|EPI_ISL_410715|2020-02-04	Singapore/38/2020|EPI_ISL_422431|2020-03-31<br>Singapore/39/2020|EPI_ISL_422432|2020-03-31<br>Singapore/40/2020|EPI_ISL_422433|2020-03-31<br>Singapore/41/2020|EPI_ISL_422434|2020-03-31<br>Singapore/42/2020|EPI_ISL_422435|2020-03-31<br>Singapore/53/2020|EPI_ISL_428832|2020-04-08<br>Singapore/54/2020|EPI_ISL_428833|2020-04-08<br>Singapore/55/2020|EPI_ISL_428834|2020-04-08<br>Singapore/56/2020|EPI_ISL_428835|2020-04-08<br>Singapore/57/2020|EPI_ISL_428836|2020-04-08<br>Singapore/58/2020|EPI_ISL_428837|2020-04-08<br>Singapore/59/2020|EPI_ISL_428838|2020-04-08<br>Singapore/60/2020|EPI_ISL_428839|2020-04-08<br>Singapore/61/2020|EPI_ISL_428840|2020-04-07<br>Singapore/62/2020|EPI_ISL_428841|2020-04-08<br>Singapore/63/2020|EPI_ISL_428842|2020-04-08<br>Singapore/64/2020|EPI_ISL_428843|2020-04-08<br>Singapore/65/2020|EPI_ISL_428844|2020-04-08<br>Singapore/66/2020|EPI_ISL_428845|2020-04-08<br>Singapore/67/2020|EPI_ISL_428846|2020-04-15<br>Singapore/68/2020|EPI_ISL_428847|2020-04-15<br>Singapore/69/2020|EPI_ISL_428848|2020-04-15<br>Singapore/70/2020|EPI_ISL_428849|2020-04-15<br>Singapore/71/2020|EPI_ISL_428850|2020-04-15
Thailand	2020-04-01	Thailand/Bangkok-0017/2020|EPI_ISL_423039|2020-03-21<br>Thailand/Bangkok-0019/2020|EPI_ISL_423040|2020-03-20<br>Thailand/Bangkok-0020/2020|EPI_ISL_423041|2020-03-21<br>Thailand/Bangkok-0021/2020|EPI_ISL_423042|2020-03-24<br>Thailand/Bangkok-0022/2020|EPI_ISL_423043|2020-03-18<br>Thailand/Bangkok-0025/2020|EPI_ISL_429164|2020-03-13<br>Thailand/Bangkok-0026/2020|EPI_ISL_429165|2020-03-13<br>Thailand/Bangkok-0028/2020|EPI_ISL_429166|2020-03-13<br>Thailand/Bangkok-0029/2020|EPI_ISL_429167|2020-03-13<br>Thailand/Bangkok-0030/2020|EPI_ISL_429168|2020-03-17<br>Thailand/Bangkok-0033/2020|EPI_ISL_429169|2020-03-17<br>Thailand/Bangkok-0034/2020|EPI_ISL_429170|2020-03-17<br>Thailand/Bangkok-0035/2020|EPI_ISL_429171|2020-03-18<br>Thailand/Bangkok-0036/2020|EPI_ISL_429172|2020-03-21<br>Thailand/Bangkok-0037/2020|EPI_ISL_429173|2020-03-21<br>Thailand/Bangkok-0038/2020|EPI_ISL_429174|2020-03-25<br>Thailand/Bangkok-0039/2020|EPI_ISL_429175|2020-03-28<br>Thailand/Bangkok-0040/2020|EPI_ISL_429176|2020-03-28<br>Thailand/Bangkok-0041/2020|EPI_ISL_429177|2020-03-28<br>Thailand/Bangkok-0042/2020|EPI_ISL_429178|2020-03-28<br>Thailand/Bangkok-0043/2020|EPI_ISL_429179|2020-03-28<br>Thailand/Bangkok-0044/2020|EPI_ISL_429180|2020-03-28<br>Thailand/Bangkok-0045/2020|EPI_ISL_429181|2020-03-28<br>Thailand/Bangkok-0046/2020|EPI_ISL_429182|2020-03-28<br>Thailand/Bangkok-0047/2020|EPI_ISL_429183|2020-03-28<br>Thailand/Bangkok-0048/2020|EPI_ISL_429184|2020-03-28<br>Thailand/NIH_2492/2020|EPI_ISL_430841|2020-03-17<br>Thailand/NIH-2982/2020|EPI_ISL_430842|2020-03-18<br>Thailand/TNIC-1889/2020|EPI_ISL_430837|2020-03-03<br>Nonthaburi/61/2020|EPI_ISL_403962|2020-01-08<br>Nonthaburi/74/2020|EPI_ISL_403963|2020-01-13	
Vietnam	2020-03-22	Vietnam/19-01S/2020|EPI_ISL_418269|2020-01-22<br>Vietnam/19-02S/2020|EPI_ISL_418267|2020-01-22<br>Vietnam/38142/2020|EPI_ISL_416427|2020-01-24<br>Vietnam/39607/2020|EPI_ISL_416428|2020-03-07<br>Vietnam/CM295/2020|EPI_ISL_416430|2020-03-06<br>Vietnam/CM296/2020|EPI_ISL_416431|2020-03-06<br>Vietnam/CM99/2020|EPI_ISL_416429|2020-02-11<br>Vietnam/VR03-38142/2020|EPI_ISL_408668|2020-01-24	

J. Microbiol. Biotechnol.
https://doi.org/10.4014/jmb.2006.06009

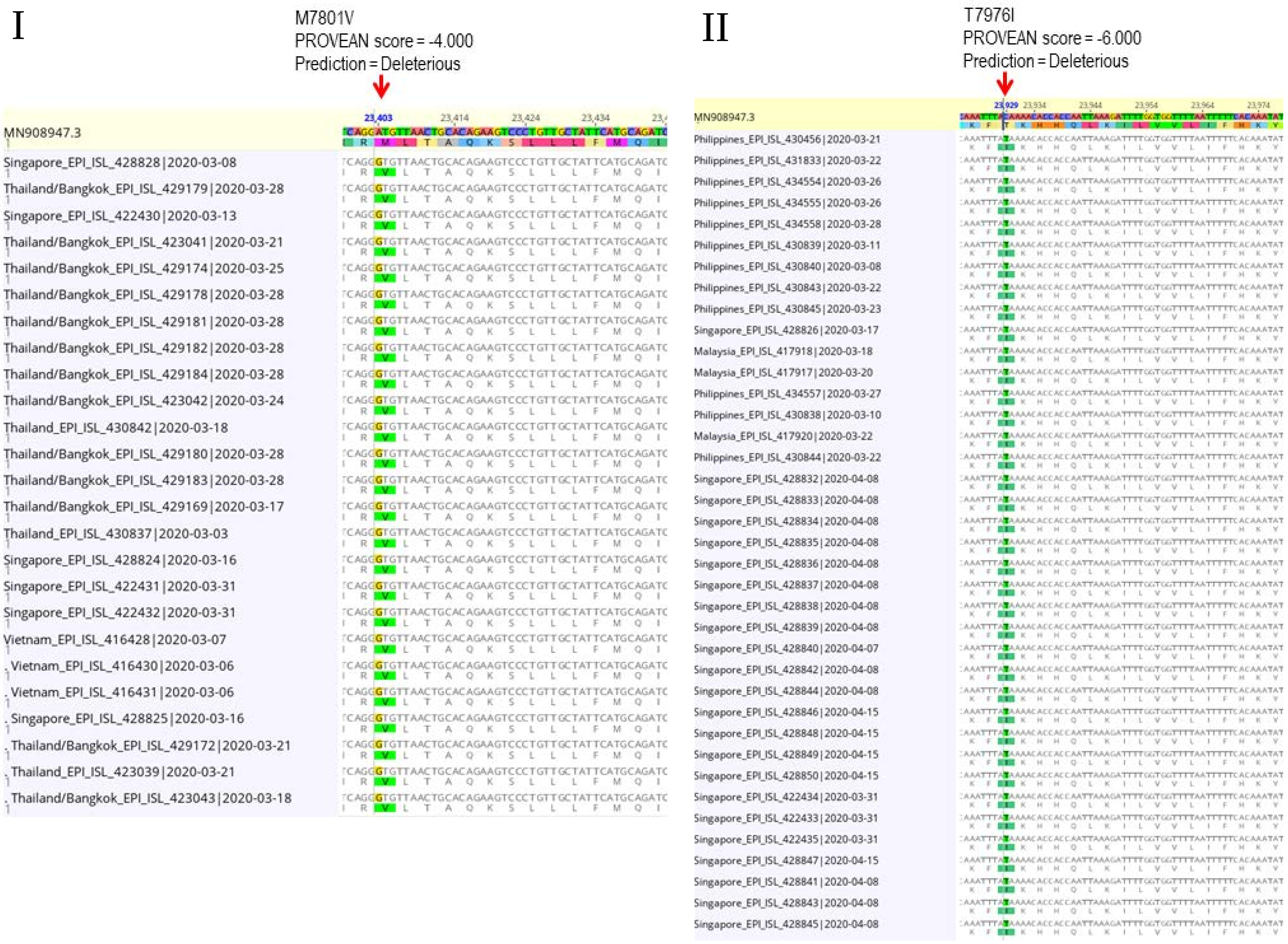

Supplementary Figure. Protein sequence chromatogram showing the I) M7801V (23403A>G) and II) T7976I (23929C>T) in the Southeast Asian SARS-CoV-2 genomes with PROVEAN prediction.
